# Age Differences in Intra-Individual Variability in Simple and Choice Reaction Time: Systematic Review and Meta-Analysis

**DOI:** 10.1371/journal.pone.0045759

**Published:** 2012-10-11

**Authors:** Dominika Dykiert, Geoff Der, John M. Starr, Ian J. Deary

**Affiliations:** 1 Department of Psychology, Centre for Cognitive Ageing and Cognitive Epidemiology, University of Edinburgh, Edinburgh, United Kingdom; 2 Medical Research Council Social and Public Health Sciences Unit, Glasgow, United Kingdom; 3 Geriatric Medicine Unit, Centre for Cognitive Ageing and Cognitive Epidemiology, University of Edinburgh, Edinburgh, United Kingdom; Cardiff University, United Kingdom

## Abstract

**Background:**

Intra-individual variability in reaction time (RT IIV) is considered to be an index of central nervous system functioning. Such variability is elevated in neurodegenerative diseases or following traumatic brain injury. It has also been suggested to increase with age in healthy ageing.

**Objectives:**

To investigate and quantify age differences in RT IIV in healthy ageing; to examine the effect of different tasks and procedures; to compare raw and mean-adjusted measures of RT IIV.

**Data Sources:**

Four electronic databases: PsycINFO, Medline, Web of Science and EMBASE, and hand searching of reference lists of relevant studies.

**Study Eligibility:**

English language journal articles, books or book chapters, containing quantitative empirical data on simple and/or choice RT IIV. Samples had to include younger (under 60 years) and older (60 years and above) human adults.

**Study Appraisal and Synthesis:**

Studies were evaluated in terms of sample representativeness and data treatment. Relevant data were extracted, using a specially-designed form, from the published report or obtained directly from the study authors. Age-group differences in raw and RT-mean-adjusted measures of simple and choice RT IIV were quantified using random effects meta-analyses.

**Results:**

Older adults (60+ years) had greater RT IIV than younger (20–39) and middle-aged (40–59) adults. Age effects were larger in choice RT tasks than in simple RT tasks. For all measures of RT IIV, effect sizes were larger for the comparisons between older and younger adults than between older and middle-aged adults, indicating that the age-related increases in RT IIV are not limited to old age. Effect sizes were also larger for raw than for RT-mean-adjusted RT IIV measures.

**Conclusions:**

RT IIV is greater among older adults. Some (but not all) of the age-related increases in RT IIV are accounted for by the increased RT means.

## Introduction

Intra-individual variability in reaction time (RT IIV)—generally understood as the variability of the responses of one individual on a single test within a single testing occasion—is often reported to increase with age in adulthood [1]–4. RT IIV is thought to be an indicator of the functioning of the central nervous system (CNS) [5]. The available empirical evidence supports this notion, in that greater RT IIV is observed in a number of conditions affecting the CNS; for example, in neurodegenerative diseases (Alzheimer's or Parkinson's Disease [6]) or following a traumatic brain injury [7], [8]. Greater RT IIV has also been reported in states that temporarily affect the CNS functioning, such as alcohol consumption [9], or presence at high altitude [10], [11]. Moreover, RT IIV is associated with white matter integrity in otherwise healthy adults [12]. Taken together, this evidence suggests that increased RT IIV marks a deterioration of the CNS functioning.

As well as marking concurrent neurological dysfunction, RT IIV has predictive validity. For example, it predicts cognitive decline over 6 years [13], progression from healthy ageing to mild cognitive impairment [14], and even mortality [15].

As we shall show, there is some evidence that RT IIV increases with age in normal healthy ageing—that is, even in adults who do not demonstrate clinical impairment. Given its predictive value, RT IIV may also be a useful screening tool for early signs of age-related neuropathology. However, there are a number of issues relating to RT IIV and ageing that remain unresolved to date and that limit the conclusions that can be drawn from the available research. Although many investigations find significant age effect on RT IIV, the magnitude of the effect varies between studies. There are also reports of there being no significant age effects in RT IIV [16], [17]. Therefore, it is desirable to investigate the issue systematically and to attempt to quantify the differences between older and younger individuals as well as to investigate potential sources of heterogeneity in the findings of various investigators.

### 1.1 Magnitude of age differences in RT IIV and the effects of procedural factors

Many studies that have investigated age effects on RT IIV have found greater levels of variability in older than younger individuals [2], [3]. However, some have not found a significant age effect on RT IIV [16]. Because RT can be obtained from a number of different tasks, it is expected that some of the differences between study findings may be due to differences in the tasks and procedures adopted by different studies. A classical RT task involves a presentation of a stimulus following which a simple response is required. However, there is great scope for variability in how the response is obtained. For example, there might be different stimuli, different intervals before their presentation, and different responses required of participants. All these might alter any age effects on RT IIV.

Considering stimuli, the main source of potential influence on the result is their mode of presentation. It is well-established that RTs differ between modalities [18]—they tend to be faster in response to auditory than visual stimuli, and intermediate for tactile stimuli. Therefore, it is possible that RT IIV of responses to stimuli in different modalities is different, and age effects on them may also be different. Another aspect of stimulus presentation which might have an effect is the length of the preparatory interval (PI); that is, the time between the beginning of an RT trial and the stimulus presentation. For example, this could be the interval between a “get ready” signal and stimulus presentation. In simple reaction time (SRT), where there is only one stimulus and one response, it is common practice to use variable PIs to minimise anticipatory responses. However, in choice reaction time (CRT) tasks, where there are a number of different stimuli presented each requiring a different response, PIs can be either variable or have a fixed length. RTs of older adults to stimuli presented following a short PI (where PIs are variable) tend to be disproportionately longer than RTs of younger adults [19]. Thus, differences in RT IIV between older and younger adults might be amplified in tasks which use variable (and short) PIs.

It has also been suggested that age effects on RT IIV tend to be larger in tasks that are more complex [20], [21]. However, the tasks that are considered “more complex” often involve executive processing to a greater extent. For example, both Dixon et al. [20] and West et al. [21] used a 1-back task, which involves retaining a stimulus presented at a previous trial in the working memory and comparing it with the current stimulus. On the other hand, Hultsch et al. 's [3] data show the opposite pattern: the RT IIV differences between younger and older groups were less marked for more complex tasks (requiring a semantic or lexical decisions) than for SRT or CRT tasks. Given these findings, it becomes apparent that some “more complex” tasks may require different cognitive mechanisms than “simple tasks” and, as such, these classes of tasks may not be directly comparable. Any comparisons of age-group differences in RT IIV from such different tasks are, at best, difficult to interpret due to multiple potential sources of any differences found.

This review will address the issue of age effects on RT IIV from tasks of different difficulty. However, the tasks considered herein will not involve complex processing or memory demands. Comparisons will be made for SRT, which requires the detection of a stimulus and response execution; and for CRT, which requires additional steps of stimulus identification and the selection of an appropriate response. In addition, the number of possible choices in the CRT task will be considered as a measure of task difficulty.

### 1.2 Different measures of RT IIV

A further issue which needed addressing was whether age differences found in RT IIV depend on the IIV measure used. There is no consensus on how to best conceptualise IIV and, consequently, several different measures are currently used. Among the simplest and most commonly-used measures of IIV is intra-individual standard deviation (ISD); that is, the standard deviation of each person's responses calculated over multiple RT trials. The standard deviation is commonly used to describe the amount of variability between subjects, so applying this statistic to within-person variability is a natural choice. The resulting measure is both easy to calculate and intuitively understandable to many. Sometimes, to avoid issues associated with aberrant responses, IIV is operationalised as a percentile difference instead. One example may be the inter-quartile range (IQR), which excludes the top and bottom quartile of RTs of each person. IQR is a special case of a family of measures based on percentile differences and, occasionally, percentiles other than 25^th^ and 75^th^ are used. For example, Adam et al., [22] used a difference between the 90^th^ and 10^th^ percentiles as an index of IIV.

One problem that affects the measures mentioned above is that mean RT and RT IIV are positively correlated, with correlations for SRT and CRT in the range of .5 to .6 [23]. Theoretical considerations of the relation between RT mean and IIV are beyond the scope of this review but, in brief, the direction of causality of the association is not well understood. An increase in mean RT may drive the increase in IIV but, the opposite may be true if some variability-producing forces are at play and it is the IIV that drives increases in mean RT. The third possibility is that mean RT and RT IIV are not causally linked at all, but instead both reflect influences from another, shared source or sources. Researchers adopt various methods in an attempt to account for the association between mean RT and RT IIV. A simple measure that controls for the mean RT is the coefficient of variation (CV), which is the ratio of ISD to the individual mean RT. Another commonly-used approach is partialling out the effects of age on individual trial RTs prior to calculating ISDs (thus obtaining what is sometimes termed “purified” residuals [3]). In this review, we will refer to the measures which take mean RT into account as “mean-adjusted” measures, and those that do not will be referred to as “raw” measures of IIV.

There are some suggestions that RT IIV increases with age only when a raw measure of IIV is used. For example, Shammi et al. [17] found a significant age difference in CRT IIV, but only when mean RT was not controlled. This finding suggests that links between older age and IIV unadjusted for RT mean may be spurious. If any increase in IIV results from general slowing of RTs, then individual differences in IIV might not be of clinical or practical importance. However, a number of researchers report significant age effects on IIV even after controlling for individual mean RT, e.g. [3], [24], [25]. Others, who use more than one measure of IIV, typically find an attenuation of effect sizes for differences in IIV adjusted for the RT mean [2]. Given the lack of consistency in findings using different measures of RT IIV, two questions remain: (1) are there age differences in IIV that are over and above age differences in mean RT?; and, if so, (2) are they similar across different measures of mean-adjusted IIV?

### 1.3 Study aims

Given the issues outlined above, the aims of this review were as follows:

1. To establish and quantify the differences in RT IIV between older and younger adults.
2. To investigate whether any differences found are more prominent in more difficult tasks (e.g., in CRT rather than SRT)
3. To investigate age-group differences in the two broad types of variability measures: raw IIV and IIV adjusted for RT mean.
4. To investigate whether and how various procedural factors (such as stimulus modality, length and variability of PI, or response type) affect the age differences found

## Methods

This systematic review has been carried out following the PRISMA guidelines [26]. The completed PRISMA checklist can be found in Appendix S1.

### 2.1 Search strategy

Searching for relevant studies was performed in two stages. First, a search of electronic databases was performed mainly in the first half of the year 2008. From this point onwards, this search will be referred to as “the main search”. Because of the large number of studies from which the full text needed reviewing, assessing eligibility of studies identified was a lengthy process. Therefore, another search was performed, aiming at including any relevant studies published between the time when the main search was performed and the end of evaluation of the studies identified through it. The second search will be referred to as “the update search.”

#### 2.1.1 The main search

Four electronic databases, PsycINFO (accessed via EBSCOhost), Medline (via OvidSP), Web of Science (via ISI Web of Knowledge), and EMBASE (via OvidSP) were searched for relevant studies from their respective inception to the date when the search was performed. PsycINFO was searched from 1806 to 4/01/08, MEDLINE from 1966 to 13/03/08, Web of Science from 1900 to 23/06/08, and EMBASE from 1980 to 31/07/08. Hand searching involved scanning the reference lists of all studies selected for inclusion. The broad aim of the search strategies was to identify studies which used a RT test and considered variability in the responses. Therefore, the general format of the strategies was as follows: (1) to identify a set of studies which used a RT test; (2) to identify a set of studies that considered IIV; and (3) to identify studies which were flagged as belonging to both these sets (i.e., those that considered IIV in an RT task). Where possible, the fields searched included title, abstract and keyword or subject heading, but these varied between databases. Full lists of search terms for each database are shown in Appendix S2.

#### 2.1.2 The update search

The same four databases were searched again for studies published in years 2008 and 2009. Consequently, all databases were searched from their inception to 31/12/2009. The search strategy was the same as before, with the exception of PsycINFO which, during the update search, was accessed via Ovid SP (access to EBSCOhost was no longer available). Again, reference lists of the relevant articles were scanned for additional studies for potential inclusion.

### 2.2 Inclusion criteria and study selection

All studies identified by the main and update searches were evaluated using the following criteria: language and publication status, study sample, availability of empirical data, RT task, and IIV measure. These are described in detail in the following sections (2.2.1–2.2.5).

Throughout the screening process, any uncertainties were discussed among all authors and consensus was sought. On no occasion was it necessary to contact authors of original papers to resolve the issues. In the case of multiple publications from a single study (on the same or related datasets) only one was selected for inclusion in the review. The primary rule used was to select the publication from which most relevant data were available.

#### 2.2.1 Language and publication status

Included studies had to be journal articles, books or book chapters published in the English language. Because there is no full listing of sources such as technical reports or unpublished manuscripts, which precludes a systematic approach to their review, they were not included.

#### 2.2.2 Study sample

Studies were included if they employed a sample of human adults, aged 18 years or above; any studies that used simulated data rather than data collected from human participants were excluded. Younger participants, aged 16 or 17, were deemed acceptable if they were included in a broader age group, for example 16 to 25. If the age of participants was not specified but a general description was provided, indicating that the sample included adults (e.g. “university students”, “sophomores”, “young adults”, or “elderly”), the criterion was considered to be met. To allow inclusion in meta-analysis, additional criteria regarding age groups and minimal sample sizes were applied: the sample had to include at least 10 older adults (aged 60 years or above) and 10 younger adults (<60 years).

#### 2.2.3 Availability of empirical data

To be considered eligible for the review, studies had to analyse quantitative empirical data. Thus, opinions, commentaries, theoretical or review papers were not included.

#### 2.2.4 RT task

The studies were included in the review if they used a qualifying SRT or CRT task. A task was considered eligible if it met all the following criteria: (1) it was a SRT or CRT task – with one or multiple possible responses, respectively; (2) it was of a stimulus-response nature, where each stimulus had a pre-determined response assigned to it (for example stimuli 1 and 2 were mapped to response buttons 1 and 2, respectively); (3) it required conscious and voluntary responses (e.g., not reflexes or responses resulting from transcranial stimulation), the latency of which could be objectively determined: for example, a button press or release; (4) it was administered as a single task (with no concurrent task being carried out as in a dual-task paradigm, for example); (5) any experimental manipulations were not related to the modality of the stimulus presentation or response (e.g. no visual degradation of stimuli in a visual RT or introducing of extraneous noise in an auditory RT task). A task was not considered eligible if it involved at least one of the following: (1) higher cognitive decision or judgement (e.g., true or false; same or different); (2) categorisation (e.g., animal/not animal); (3) executive function (e.g., inhibiting a response in a go/no-go or stop-signal task); (4) memory (e.g., in an n-back task).

#### 2.2.5 IIV measure

To be eligible, a study needed to consider RT IIV across trials within a single testing occasion. There was no restriction in terms of the measure of IIV used; any measure claimed by the study's authors to reflect within-subject variability was accepted. However, measures of IIV calculated across occasions or across different tasks were not eligible for this review.

### 2.3 Quality assessment

Quality assessment of the included studies comprised evaluation of the sample representativeness and data treatment. The degree of sample representativeness was assessed depending on the sampling procedure and coded as representative (random sampling or whole population sampled), likely to be representative (systematic or stratified sampling), and not likely to be representative (non-probability sampling, e.g. purposive, convenience, or snowball). If insufficient information was provided in the paper, a judgement was made based on the sample size and the likelihood that the sample was purposive or convenience.

Another aspect of study quality assessment involved the assessment of data treatment. Specifically, it was considered whether or not trial-level trimming was performed. Trial-level trimming usually includes eliminating extremely fast and slow responses, which are likely to result from accidental key presses or a distraction or lapse in concentration, respectively. Excluding such aberrant responses prior to estimating RT IIV improves the reliability of the measure.

### 2.4 Data extraction

Data from the included studies were extracted using a specially-designed data extraction form. For each study, information about the participants, tasks and RT IIV data for each age group of interest were extracted. Detailed information on tasks were collected, including the number of trials, stimulus type and modality, PI, etc., for later use as possible moderating variables.

The aim of this review was to compare RT IIV performance between older (age 60 or above) and younger adults (aged under 60). During the pilot data extraction, it became apparent that there were considerable differences in the younger group age ranges among studies. Therefore, the younger (under 60) group was further subdivided into young and middle-aged groups. To avoid overlap between groups, restrictions were imposed such that the boundaries (with 1 year tolerance) were, 16 to 39 for young, 40 to 59 for middle-aged, and 60 years and above for old (no upper limit was defined for the old group).

Where no age groups were created in the publication, the authors were contacted and asked to provide data for these groups. If the sample in a study was already subdivided into age ranges, then the relevant groups were selected. The criteria for selecting an age group were as follows. Groups which were contained in the selected age ranges were selected over those that crossed the boundaries. If more than one group was contained within a single age range, then the one closest to or containing age 25, 45, or 65 (for young, middle-aged, and old, respectively) was selected. For the youngest group, ranges excluding ages of below 18 years were given priority over those including teenagers.

The measure of interest in the present review was RT, defined as time elapsed between a stimulus to elicit a pre-determined response and the execution of this response. However, occasionally, rather than the overall RT, authors report RTs fractionated into decision (DT) and movement (MT) components; for example, when a response involves releasing a home key and pressing a response key [27]. For these studies, DT rather than MT was selected, as it is intended to capture the time taken to complete the cognitive, rather than motor, component of the task.

All authors who provided only one type of IIV measure (raw or mean-adjusted) were contacted and asked to provide the other. We also contacted authors of studies from which no IIV data were reported, but where it was implied in the report that they have been collected and/or considered (for example, when RT distributions were considered, [28]). To assist comparability across studies, whenever contacting authors, the most commonly used raw and mean-adjusted measures were requested: RT ISD and CV, respectively.

If an author could not be contacted or the original data were not available, the paper was screened for other usable data sources. For studies which reported significance tests for comparisons between two groups, effect sizes were estimated from these statistics using conversion formulae provided by Wolf [29]. This was done for three studies ([17], [28], and [30]). If the relevant data were only presented graphically, the graphs were digitised using Engauge Digitizer software (version 2.15). Each graph was digitised twice and the values obtained from each were averaged to form more reliable estimates. Digitisation of graphs was performed for four studies ([30], [31], [32], and [33]). The principal summary measure was Cohen's *d* for older-younger difference.

## Results

**Figure 1:**
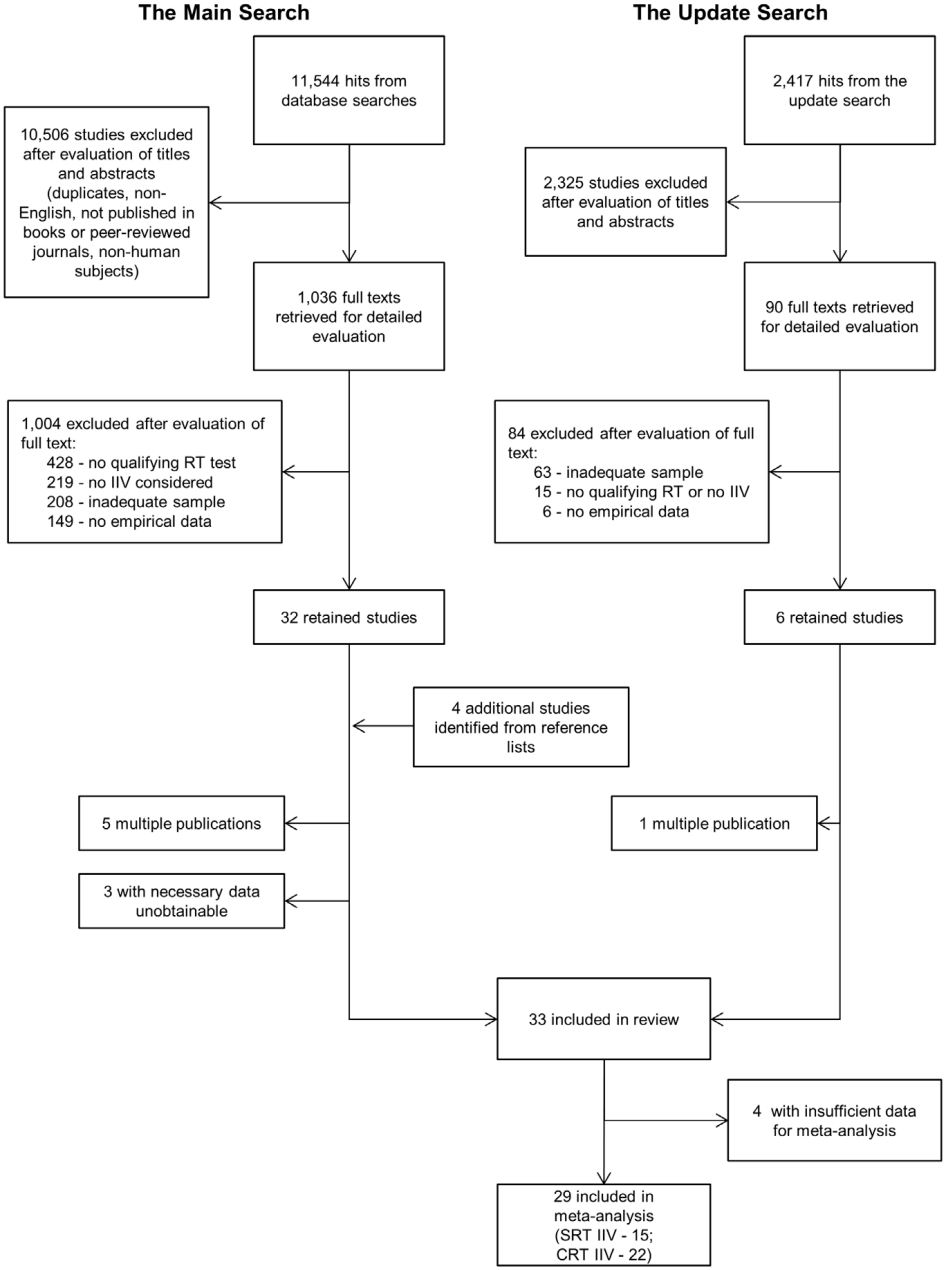
Flowchart of the review process.

Figure 1 illustrates the flow of studies through the review process. Titles and abstracts identified by the main search (n = 11,544) were screened for relevance using the inclusion criteria specified earlier. If the title and abstract did not provide sufficient information to justify exclusion of a study, a full text was obtained for further screening. Overall, 1,036 full texts were retrieved for detailed consideration, out of which 1,004 were deemed not relevant and excluded: 428 did not use a qualifying RT test, 219 did not report data on IIV, 208 did not have an adequate sample (e.g. non-human, children, fewer than 10 people aged over 60), and 149 did not report any empirical data. Thirty two studies were retained for further review. Four additional relevant studies were identified through hand searching of reference lists of the retained papers. Five studies reported on data that overlapped with other studies included in the review, and were therefore excluded. A further three studies were excluded because the data necessary for them to be included in the review were unobtainable. These exclusions left 28 studies to be reviewed.

The update search resulted in 2,417 hits and titles and abstracts of these were screened for relevance. In total 90 full texts were obtained for detailed review, and 84 did not meet some of the inclusion criteria (63 were excluded because of inadequate sample, 15 had no qualifying RT test or no IIV data, and 6 did not report empirical data). Six studies were retained, of which one reported data which overlapped with another paper already included in the review. Therefore, five eligible studies identified in the update search were included in the review. Reference lists of these papers revealed no additional studies eligible for inclusion.

In total, 33 studies were included in the review [2], [3], [17], [21]–[25], [27]–[28], [30]–[52]—18 with data on SRT IIV and 24 with data on CRT IIV. Of these, 29 provided sufficient data for inclusion in meta-analysis (SRT IIV, n = 15; and CRT IIV n = 22). Details of the samples and age groups of all included studies are presented in Table 1. The proportion of males and females in younger and older age groups did not differ systematically across studies. Among studies that reported gender composition of age groups, 7 had greater proportion of females in the younger group and 7 had greater proportion of females in the older group, whilst the majority (n = 10) had either identical or very similar gender split in their younger and older groups.

**Table 1:** Sample characteristics of studies included in the review of simple and choice reaction time intra-individual variability studies.

Study	Sex	Representa-tive?	Young group		Middle-aged group		Old group		Review in which included
			N	Mean age (range)	N	Mean age (range)	N	Mean age (range)	
Adam et al. (2006)	M	Not likely	12	25.2 (21–31)	-	-	11	66.4 (61–70)	SRT
Anstey et al. (2005)	M,F	Likely	2,404	NR (20–24)	2,530	NR (40–44)	2,551	NR (60–64)	SRT, CRT
Bherer et al. (2006)	M,F	Not likely	12	20 (NR)	-	-	12	70 (NR)	CRT
Bunce et al. (2004)	NR	Not likely	24	25.5 (20–30)	-	-	24	69.3 (60–85)	CRT
Bunce, Handley & Gaines (2008)	M,F	Not likely	77	23.9 (18–30)	38	45.3 (41–50)	96	71.1 (61–80)	SRT, CRT
Bunce, Tzur et al. (2008)	M,F	Not likely	54	22.7 (18–30)	28	45.8 (41–50)	34	64.9 (61–70)	CRT
Deary & Der (2005)	M,F	Likely	658	Approx 24 (23–26)	741	Approx 44 (39–50)	696	Approx 63 (62–66)	SRT, CRT
Der & Deary (2006)	M,F	Likely	1,706	24.1 (18–30)	1,341	44.7 (40–50)	1,563	67.9 (60–80)	SRT, CRT
Duchek et al. (2009)	NR	Not likely	35	20.29 (NR)	-	-	220	71.75 (NR)	CRT
Finkel & McGue (2007)	M,F	Likely	35	32.8 (27–35)	40	44.4 (40–50)	175	66.0 (60–80)	SRT, CRT
Fontani et al. (2004)	M,F	Not likely	17	25.0 (18–29)	17	51.0 (46–57)	17	66.0 (61–77)	SRT
Fozard et al. (1976)	M	Not likely	24	Median = 34 (25+)	24	Median = 50	24	Median = 69	CRT
Fozard et al. (1994)	M,F	Not likely	226	NR (25–34)	187	NR (35–44)	232	NR (65–74)	SRT
Gooch et al. (2009)	M,F	Not likely	16	22.6 (19–29)	-	-	16	72.8 (62–81)	CRT
Gorus et al. (2006)	M,F	Not likely	27	28.5 (19–37)	-	-	27	74.7 (64–84)	SRT, CRT
Hogan (2003)	M,F	Not likely	78	18.8 (NR)	-	-	94	70.1 (60+)	SRT, CRT
Hultsch et al. (2002)	M,F	Not likely	99	23.2 (17–36)	-	-	361	69.6 (65–74)	SRT, CRT
Li et al. (2009)	M,F	Likely	25	25.3 (18–30)	26	45.6 (40–50)	68	70.5 (60–80)	CRT
Martin et al. (2009)	M	Not likely	-	-	29	44.4 (39–50)	39	63.7 (61–70)	CRT
McAuley et al. (2006)	M,F	Not likely	43	19.6 (17–22)	-	-	33	72.9 (61–82)	CRT
Obrist (1953)	M	Not likely	25	27.5 (18–39)	-	-	57	71.5 (65–75)	SRT
Pierson & Montoye (1958)	M	Not likely	60	NR (19–30)	40	NR (41–55)	40	NR (66–85)	SRT
Rakitin et al. (2006)	M,F	Not likely	31	24.4 (18–35)	-	-	32	71.2 (60–86)	CRT
Shammi et al. (1998)	F	Not likely	18	27.8 (20–35)	-	-	18	68.2 (60–75)	CRT
Smulders (1997)	M	Not likely	12	20.7 (18–24)	-	-	12	66.8 (62–73)	CRT
Sparrow et al. (2006)	M	Not likely	10	26.3 (20–32)	-	-	10	71.1 (64–78)	SRT
Spirduso & Clifford (1978)	M	Not likely	15	22.2 (20–30)	-	-	15	64.2 (60–70)	SRT, CRT
Surwillo (1963)	M	Not likely			N/A	40 (N/A)	N/A	60 (N/A)	SRT
West et al. (2002)	NR	Not likely	20	23.9 (19–29)	-	-	20	73.8 (65–83)	CRT
Wilkinson & Allison (1989)	M,F	Not likely	1,189	NR (20–29)	208	NR (40–49)	50	NR (60–69)	SRT
Williams et al. (2005)	M,F	Not likely	47	NR (18–29)	28	NR (45–59)	25	NR (60–81)	CRT
Williams et al. (2007)	M,F	Not likely	80	24.8 (20–29)	93	45.2 (40–49)	27	66.6 (60–76)	CRT
Yan et al. (1998)	M,F	Not likely	20	24.4 (20–30)	-	-	20	70.4 (65–80)	SRT

*Note*. Numbers of participants reflect actual numbers used to obtain estimates of intra-individual variability and may differ from those reported in papers.

M = males, F = females, NR = not reported, SRT = Simple Reaction Time, CRT = Choice Reaction Time.

In section 3.1 we present the results for SRT IIV in the following order: raw values for old versus young, then old versus middle-aged; followed by mean-adjusted values for old versus young and old versus middle-aged; then an assessment of the attenuation due to adjusting for RT mean, and finally, evidence from studies not included in any meta-analyses. Section 3.2 repeats this for CRT IIV, but adds a penultimate subsection on the number of choices. Section 3.3 assesses publication bias.

### 3.1 SRT IIV

Eighteen studies contributed SRT IIV data to this review and the details of their tasks and data are summarised in Table 2 (see also Table 1 for information on age groups). The studies differed in their measurement of RT in a number of ways. In terms of stimulus modality, most studies used a visual mode of stimulus presentation. The visual stimuli used varied between studies, but were usually static; i.e., they appeared at the beginning of a trial and remained unchanged until a response was made. Commonly-used stimuli were a light (n = 5), letter (n = 3), shape (n = 2), or digit (n = 2). Two studies used a dynamic stimulus, in the form of a timer which started at the beginning of a trial and continued to increment until a response was made. Four studies used auditory stimuli (i.e., tones or a buzzer sound). Gorus et al. [45] tested both visual and auditory SRTs and data on the former were selected in keeping with the majority of the remaining studies. Most of the responses to the stimuli in the reviewed studies involved either pressing a response key or releasing a home key. In one study that differed from the others, participants were asked to move a hand-held stylus repeatedly between two target circles [52]. The RT was measured between the “go” signal and the initiation of the back-to-front movement series.

**Table 2:** Summary of task characteristics and data available from simple reaction time intra-individual variability studies.

Study	Modality	Stimulus	PI: variable/fixed, values (s)	Trials: test (practice)	Response	Data source	Trial-level trimming	Raw IIV measure	Adjusted IIV measure
Adam et al. (2006)	Visual	Timer	Variable, 2,000–10,000	Approx. 100 (NR)	Pressing a response key	Provided by author(s)	Yes - Percentile difference IIV measure eliminates extreme RTs	90th-10th percentile	90th-10^th^ percentile/mean RT
Anstey et al. (2005)	Visual	Light	NR	80 (NR)	Pressing a response key	Provided by author(s)	Performed	ISD	Mean-independent variability
Bunce, Handley & Gaines (2008)	Visual	Letter	Variable, 300–1,000	48 (8)	Pressing a response key	Provided by author(s)	Performed	ISD	Purified ISD
Deary & Der (2005)	Visual	Digits	Variable, 1,000–3,000	20 (8)	Pressing a response key	Provided by author(s)	Not performed (trial data not available)	ISD	CV
Der & Deary (2006)	Visual	Digit	Variable, 1,000–3,000	20 (8)	Pressing a response key	Provided by author(s)	Not performed (trial data not available)	ISD	CV
Finkel & McGue (2007)	Visual	Light	Variable, approx. 5,000	15 (3)	Releasing a home key and pressing a response key (RT = time to release, DT)	Provided by author(s)	Performed	ISD	CV
Fontani et al. (2004)	Visual	Letter	Variable, 2,000–4,000	80 (5 minutes)	Pressing a response key	Reported in paper	Performed	-	VI = ISD/(1000/mean RT)
Fozard et al. (1994)	Auditory	Tones	Variable, 6,000–13,000	20 (46)	Pressing a response key	From a Master's thesis of a co-author	Performed	Variance	Variance/mean
Gorus et al. (2006)	Visual (Auditory also available but not used here)	Light	Variable, 3,000–6,000	28 (NR)	Releasing a home button and pressing a response button (RT = DT+MT used)	Provided by author(s)	Performed	IQR	IQR/MD*100
Hogan (2003)	Visual	Square	Variable, 1,000–3,000	Approx. 33 (NR)	Pressing a response key	Raw: reported in paper; adjusted: digitised graph	Performed	MD ISD	MD ISD adjusted for MD RT
Hultsch et al. (2002)	Visual	Plus sign	Variable, 500–1,000	50 (NR)	Pressing a response key	Provided by author(s)	Performed	ISD	Purified ISD
Obrist (1953)	Auditory	Blended click and buzzer sound	Variable, 1,000–2,000	50 (25+)	Pressing a response key	Reported in paper	Yes - Semi IQR eliminates extreme RTs	Semi IQR	-
Pierson & Montoye (1958)	Visual	Light	NR, Approx. 2,000	15 (15)	Releasing a key (DT)	Digitised graph	Frequency of mode disregards extreme RTs	Mean frequency of mode	-
Sparrow et al. (2006)	Visual	Letter	Variable, 6,000–18,000	90 (NR)	Pressing a response key	Digitised graph	Performed	ISD	CV
Spirduso & Clifford (1978)	Visual	Light	Fixed, 20,000	50 (NR)	Releasing a home key and press a response key (DT used here)	Reported in paper	NR	Mean of ISD across 5 blocks of 10 trials	-
Surwillo (1963)	Auditory	Tone	Variable, 10,000–25,000	Approx. 20 (NR)	Pressing a response key	Estimated from a regression equation	NR	ISD	-
Wilkinson & Allison (1989)	Visual	Timer	Variable, 1,000–10,000	8 (2)	Pressing a response key	Digitised graph	Performed	-	CV
Yan et al. (1998)	Auditory	Tone	Variable, 150–1,000	10 (5)	Beginning of a back to front movement series; removing stylus from a home position	Reported in paper	NR	ISD	-

Note. CV = coefficient of variation, IIV = intra-individual variability, IQR = inter-quartile range, ISD = intra-individual standard deviation, MD = median, NR = not reported, PI = preparatory interval, VI = Variability index.

Given that SRT does not require a response selection, presenting stimuli at regular intervals may elicit anticipatory responses. Not surprisingly, most SRT studies used variable PIs. However, this could not be determined for three studies. The length of PIs varied greatly across studies; the shortest PI was 150 ms and the longest 25 s (overall median = 3 s). The number of trials per study varied from 8 to 100 (median = 30.5).

The obtained raw SRT IIV measures were either dispersion around the individual mean, (i.e., ISD or variance; n = 12) or percentile difference, such as inter-quartile range (IQR; n = 3). Adjusted SRT IIV measures were either coefficient of variation (CV; six based on ISD, e.g., ISD/mean RT or ISD/median RT; and two based on percentile differences, e.g., IQR/mean RT) or variability index (n = 1), VI = ISD/(1000/mean RT). Three studies used a regression method to control for RT mean differences and calculated ISD on the “purified” residuals. Hogan [30] also used regression method to control for central tendency in RT (median in this case), but did not calculate ISD from residuals. Instead, *R^2^* from regression models that included age and median RT were used to estimate RT IIV differences between age groups. Finally, one study provided a crude measure of the frequency of mode (explained in detail in section 3.1.6), which was then averaged across participants in each age group [31].

Out of the 18 studies included in the review, 15 had sufficient data to allow their inclusion in the meta-analysis: 13 provided data on raw SRT IIV, and 13 provided data on SRT IIV adjusted for mean SRT. Sufficient data were not available from three studies and they could not be included in the meta-analysis [27], [31], and [51]. These studies and their findings are briefly summarised in section 3.1.6. The remaining SRT IIV studies contributed data to four meta-analyses: raw SRT IIV in old versus young participants, adjusted SRT IIV in old versus young participants, raw SRT IIV in old versus middle-aged participants, and adjusted SRT IIV in old versus middle-aged participants.

Heterogeneity between studies was assessed in each meta-analysis using Cochran's Q test. There was significant heterogeneity among studies in all four comparisons (all *p*s<.001; *I* ^2^ range 74.89 to 87.70); therefore, random effects method was applied to pool effect sizes.

#### 3.1.1 Raw SRT IIV: old versus young

**Figure 2:**
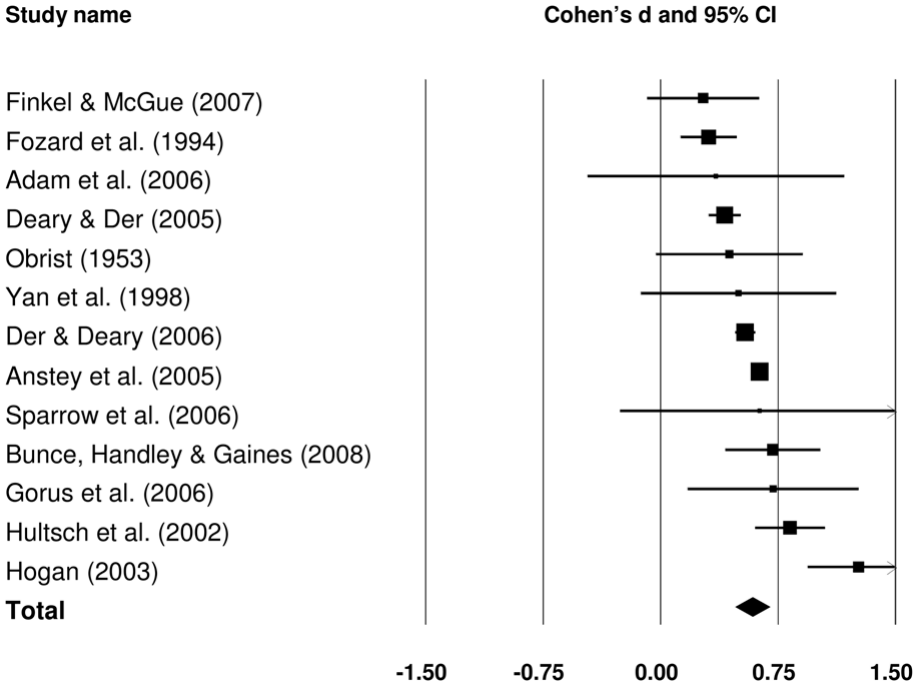
Effect sizes for old versus young comparisons in raw simple reaction time intra-individual variability studies.

Effect sizes for the old-young group difference in raw SRT IIV could be obtained from 13 studies. The forest plot in Figure 2 presents effect sizes (Cohen's *d*) for all old-young comparisons. To evaluate the magnitude of the effect sizes, we used values of 0.20 for a small effect, 0.50 for a medium effect, and 0.80 for a large effect, as suggested by Cohen [53]. The pooled effect size was medium in magnitude, *d = *0.582 (*Z = *10.220, *p*<.001), and indicated that raw IIV was larger in older than in younger groups.

To investigate possible sources of between study heterogeneity, subgroup and meta-regression analyses were performed. The groups were defined based on sample size (small: <100, medium: 100–1000, large: >1000), stimulus modality (auditory vs. visual), and two measures of study quality: sample representativeness (likely to be representative vs. not likely), and trial-level data trimming (performed vs. not performed). The effect sizes for each subgroup are presented in Table 3. Given that the number of studies in subgroups was generally small, *τ* ^2^ (the true variance between studies) was estimated for each subgroup separately and later pooled. This procedure does not reflect the assumption that variance between studies is the same for all subgroups, but is used to overcome the imprecision in the estimate of *τ* ^2^ that is likely in subgroups comprising a small (<5) number of studies [54].

**Table 3:** Summary of subgroup analysis results for simple reaction time intra-individual variability studies.

Groups compared	Raw SRT IIV				Mean-adjusted SRT IIV			
	N	ES	Z	p	N	ES	Z	p
All studies	13	0.582	10.220	<.001	13	0.370	4.960	<.001
Sample size				.584^a^				.073^a^
<100	5	0.531	3.328	.001	4	0.251	1.250	.211
100–1000	5	0.657	7.095	<.001	5	0.543	5.284	<.001
>1000	3	0.531	5.859	<.001	4	0.231	2.371	.018
Sample representativeness				.149^a^				.002^a^
Not likely	9	0.669	7.990	<.001	9	0.541	6.334	<.001
Likely	4	0.499	6.049	<.001	4	0.156	1.714	.086
Data trimming				.253^a^				.323^a^
Not performed	3	0.479	4.290	<.001	2	0.203	1.029	.303
Performed	10	0.632	8.449	<.001	11	0.423	4.109	<.001
Stimulus modality				.062^a^				.710^a^
Auditory	3	0.362	2.815	.005	1	0.287	1.197	.231
Visual	10	0.627	10.488	<.001	12	0.381	4.689	<.001
Mean-adjusted IIV measure								.199^a^
CV (based on variance or SD)	-	-	-	-	6	0.268	2.014	.044
CV (based on percentile difference)	-	-	-	-	2	-0.003	-0.009	.993
ISD on residuals purified of mean	-	-	-	-	3	0.653	3.665	<.001
Other	-	-	-	-	2	0.464	1.751	.080

Note. SRT IIV = simple reaction time intra-individual variability, CV = coefficient of variation, ES = effect size (Cohen's d), ISD = intra-individual standard deviation, PI = preparatory interval.

a p value for overall between subgroup heterogeneity.

Sample size or sample representativeness did not explain much between study heterogeneity (*p* = .584 and .149, respectively), although effects were slightly larger in less representative samples. Effect sizes were also a little larger in the subgroup of studies which adopted trial-level trimming, but not significantly so (*p = *.253). The effects tended to be slightly larger in studies in which the stimuli were presented visually rather than aurally (*p = *.062).

Meta-regression was performed on three continuous variables: age of the older group, the number of trials within the test, and the length of PI (see Figure S1). Age of the older group was mean, median or midpoint of ages for the group, depending on which measure was available. For studies in which PI was fixed, the exact value of PI was used; where PIs were variable, the median or the middle value was used.

For studies which considered raw SRT IIV, effect sizes were larger for older old groups (*B = *0.031, *se = *0.015, *p = *.042), and smaller with longer PIs (*B = *−0.040, *se = *0.019, *p = *.034). There was no significant relationship between effect size and the number of SRT trials.

#### 3.1.2 Raw SRT IIV: old versus middle-aged

**Figure 3:**
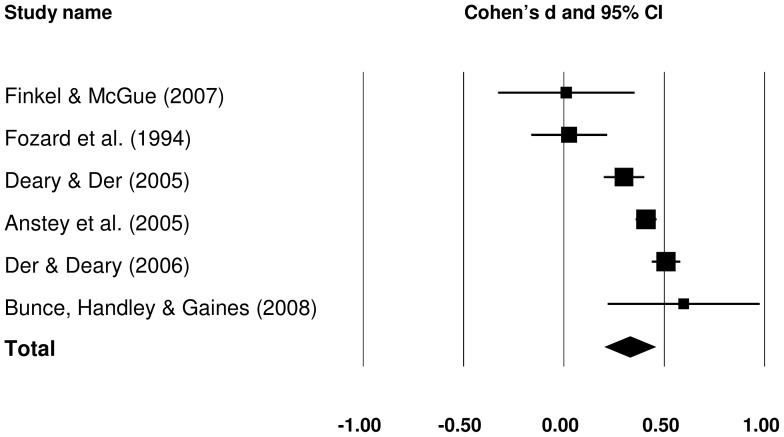
Effect sizes for old versus middle-aged comparisons in raw simple reaction time intra-individual variability studies.

Six studies provided data which contributed to the old versus middle-aged comparisons of raw SRT IIV (see Figure 3). When older and middle-aged groups were compared, the former group had greater raw SRT IIV, and the overall effect size was small, *d = *0.327 (*Z* = 5.002, *p*<.001). The modest number of studies for this comparison did not permit an investigation of sources of heterogeneity.

#### 3.1.3 Mean-adjusted SRT IIV: old versus young

**Figure 4:**
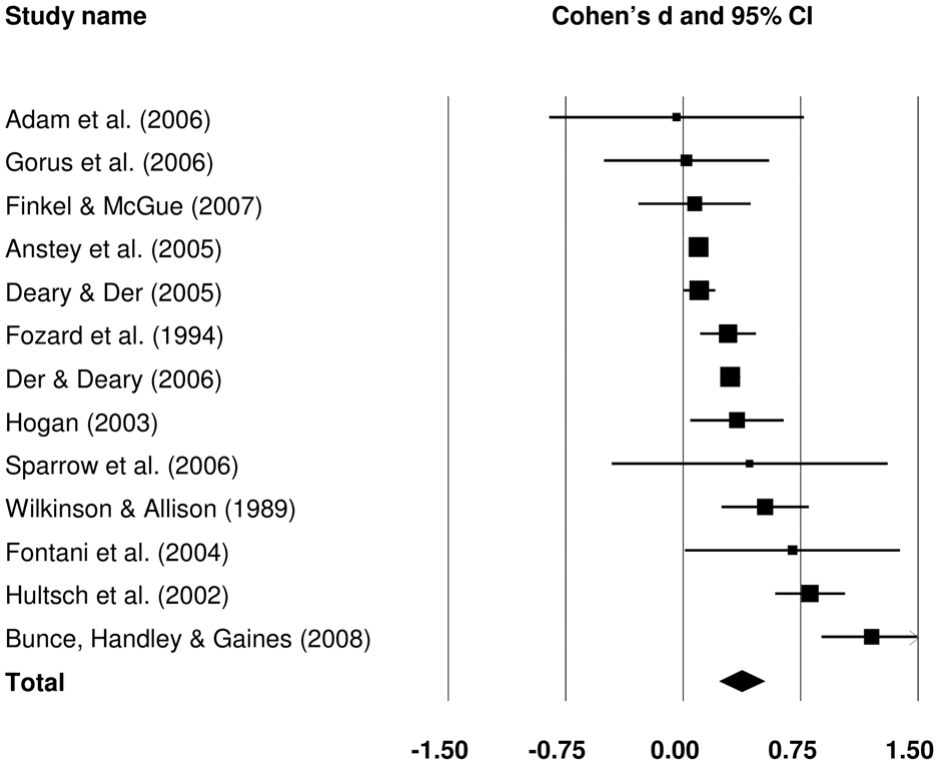
Effect sizes for old versus young comparisons in mean-adjusted simple reaction time intra-individual variability studies.

Thirteen studies considered old-young differences in SRT IIV adjusted for mean SRT and provided sufficient data to allow meta-analysis (see Figure 4). The overall effect size for the comparison was small, *d = *0.370 (*Z = *4.960, *p*<.001). It was in the expected direction, with older adults demonstrating greater variability than younger adults, even when the differences in mean SRT were controlled.

We performed subgroup analyses, as we did with raw SRT IIV, using sample size, modality, representativeness and trial-level data trimming. In addition, the measure of mean-adjusted IIV was considered, with four groups: CV based on variance or SD (e.g. ISD/mean); CV based on percentile difference (e.g. IQR/mean); ISD calculated from residuals purified of influences of mean SRT; and other, for measures which did not fall in either of the three categories. *τ* ^2^ were pooled across subgroups to reduce the imprecision of within subgroup heterogeneity estimates where the number of studies is small. The effect sizes for the subgroups considered are given in Table 3.

Studies with medium sample sizes tended to produce largest effect sizes (*p* = .073 for overall between group heterogeneity). Both small and large studies had lower estimates, and the effect size estimate for the <100 subgroup was not statistically significant. The difference between older and younger individuals appeared to be larger in studies with less representative samples, and the effect was not significant in the subgroup with more representative samples. The effect sizes from representative and non-representative samples were significantly different from each other, *p = *.002. In terms of trial-level data trimming, the effect sizes were a little larger for studies that adopted some form of trimming than those that did not. However, this difference did not reach statistical significance (*p* = .323). There was no significant difference in effect sizes between studies presenting visual stimuli and a study (n = 1) with an auditory presentation (*p* = .710), although the effect size was larger for visual presentation. Finally, when subgroups based on the SRT IIV measure were considered, it appeared that effect size was smaller for CV (based on ISD or variance) than for the ISD calculated from residuals purified of mean RT. Effect size for CV which used a percentile difference divided by individual mean was close to 0 and not statistically significant. However, the overall differences between subgroups based on SRT IIV measures were not statistically significant (*p* = .199).

Meta-regression was performed with mean age of the old group, number of trials, and PI. Effect sizes were larger for older groups with higher mean age (*B* = 0.042, *se* = 0.019, *p* = .025); the number of SRT trials and PI were not significantly related to effect sizes.

#### 3.1.4 Mean-adjusted SRT IIV: old versus middle-aged

**Figure 5:**
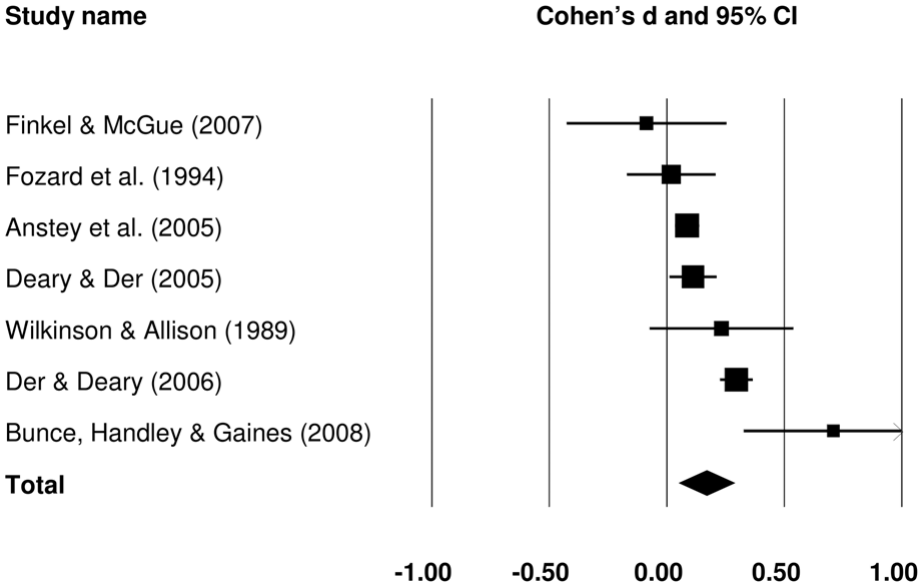
Effect sizes for old versus middle-aged comparisons in mean-adjusted simple reaction time intra-individual variability studies.

A comparison of SRT IIV adjusted for SRT mean was possible for seven studies (see Figure 5). The pooled effect size was small (*d* = 0.167, *Z* = 2.745, *p*<.001) and again the direction was as expected; that is, older groups showed more IIV than younger groups. Subgroup analyses or meta-regression were not performed for this comparison, due to a small number of studies included.

#### 3.1.5 Attenuation of age group difference in effect sizes by adjusting SRT IIV for SRT mean

Having carried out the four meta-analyses that examined age differences in SRT IIV, a pattern emerged in which effect sizes appeared to be larger for older than younger groups, and larger if SRT IIV was not adjusted for SRT mean. In order to estimate the degree to which effect sizes are attenuated by adjusting SRT IIV measures for mean SRT, pooled estimates were obtained from studies that contributed data to both analyses within each age-group comparison. In other words, meta-analyses were re-run for both old versus young and old versus middle-aged groups, but only on a subset of studies that had usable data on both raw and mean-adjusted SRT IIV. There were 11 such studies which considered old versus young differences and six studies that considered old versus middle-aged differences. When comparing older with younger groups, effect sizes are attenuated by 44.8% by using a SRT IIV measure that is adjusted for mean SRT (raw SRT IIV *d = *0.592; mean-adjusted SRT IIV *d* = 0.327). For old versus middle-aged differences the attenuation of effect sizes was 55.1% (raw SRT IIV *d* = 0.343; mean-adjusted SRT IIV *d* = 0.161).

#### 3.1.6 Evidence from SRT studies not included in meta-analyses

A relatively early study [31] used an unusual measure of SRT IIV, namely, frequency of mode. This simple measure reflects the consistency of responding, with higher values reflecting less variability. Data were only presented graphically and the estimates were obtained from a graph. Authors themselves report that IIV decreases until the age of about 30, followed by an increase. The mean frequencies of mode obtained from the digitised graph revealed that they were largest in the youngest group (4.52), smallest in the oldest group (1.97) and intermediate in the middle-aged group (3.12). Thus consistency was lower in the old group than either the young or middle-aged group, and the difference was most marked for the young-old comparison.

Spirduso and Clifford [27] reported mean SRT ISD of younger and older participants who were either physically active (racketball players or runners, with a history of training four times a week) or not active (never engaged in any sports on a regular basis). In the non-active participants SRT ISDs were slightly lower for the young (32 ms) than the old group (38 ms). However, the difference between active and non-active groups was much greater than between younger and older groups, with the latter not statistically significant.

In the final study which considered age and SRT IIV but was not included in the meta-analysis, SRT ISD correlated positively with age, with a small effect size (*r* = .26) [51]. The correlation coefficient reported was not used in the meta-analysis because it was calculated for the age range crossing the age group boundaries defined for this review. Consequently, it would not be possible to obtain an estimate of an effect size for old versus middle-aged difference without it being influenced by younger subjects in the sample. Instead, a regression equation provided by the author was used to calculate predicted ISD values at ages of 40 and 60 years (lowest ages in the ranges for our middle-aged and old groups, respectively). No estimate was obtained for age 20 years (i.e. the young group) because it was outside the age range of the sample used in Surwillo's study (28–99). The obtained values were 26.49 and 30.09, indicating that IIV of adults aged 60 was 3.60 ms greater than IIV of adults 20 years younger.

### 3.2 CRT IIV

The review process identified 24 studies with CRT IIV. Their task characteristics and data obtained from them are summarised in Table 4 (information about participants and age groups are presented in Table 1). All studies used visual stimuli, but one used both visual and auditory presentation [35]. Stimuli differed between studies. The most frequently used stimuli were lights (n = 7), digits (n = 5), letters (n = 5), and circles (n = 4). Three studies used stimuli indicating direction: arrow [30], [39] and fish (like an arrow, but adapted to make the task more appealing to children [25]. In most studies, responses involved either pressing a response key or releasing a home key (n = 23). One study used an unusual response type, with two keys pressed in order determined by the stimulus (e.g. LR required pressing the left key first, followed by the right key) [17]. Concerning the PI, most CRT tasks had a fixed PI (n = 14), with only six studies using a variable PI; three studies did not report the information and one used both fixed and variable PIs. PIs ranged from 0 (immediate stimulus onset following a response) to 20s, with an overall median of 800 ms. The number of trials per study ranged from 15 to 513 trials, with a median of 40.

**Table 4:** Summary of task characteristics and data available from choice reaction time intra-individual variability studies.

Study	Modality	Stimulus	PI: variable/fixed, values (s)	Trials: test (practice)	Number of choices, spatial/non-spatial, task objective	Response	Data source	Trial-level trimming	raw IIV measure	adjusted IIV measure
Anstey et al. (2005)	Visual	Light	NR, NR	40 (NR)	2, spatial, identify location	Pressing a response key	Provided by author	Performed	ISD	Mean-independent variability
Bherer et al. (2006)	Visual and auditory	Letter/tone	Fixed, 500	20 (probably 0, as data from a baseline session)	2, non-spatial, discrimination (B or C; high or low tone)	Pressing a response key	Provided by author	Performed	ISD	CV
Bunce et al. (2004)	Visual	Light	Fixed, 0 (pressing of home key initiated next trial)	100 (20)	2 4 8, spatial, Location of stimulus light	Pressing a response key	Reported in paper	Performed	ISD	Purified ISD
Bunce, Tzur et al. (2008)	Visual	Circle	NR, NR	100 (20)	2,4, spatial, identify location	Pressing a response key	Provided by author	Performed	ISD	Purified ISD
Bunce, Handley & Gaines (2008)	Visual	Circle	Fixed, 500	48 (12)	2,4, spatial, identify location	Pressing a response key	Provided by author	Performed	ISD	Purified ISD
Deary & Der (2005)	Visual	Digits	Variable, 1,000–3,000	40 (8)	4, non-spatial, discriminate between digits (1,2,3,4)	Pressing a response key	Provided by author	Not Performed (trial data not available)	ISD	CV
Der & Deary (2006)	Visual	Digit	Variable, 1,000–3,000	40 (8)	4, non-spatial, discriminate between digits (1,2,3,4)	Pressing a response key	Provided by author	Not Performed (trial data not available)	ISD	CV
Duchek et al. (2009)	Visual	Arrow	Fixed, 500	40 (4)	2, non-spatial, identify the direction of arrow	Pressing a response key	Provided by author	NR	-	CV
Finkel & McGue (2007)	Visual	Light	Variable, approx 5,000	15 (3)	4, spatial, discriminate between colours/locations	Releasing a home key and pressing a response key (RT = time to release, DT)	Provided by author	Performed	ISD	CV
Fozard et al. (1976)	Visual	Light	Fixed, 3,000	121 (0)	2, spatial, discriminate between colours/locations	Releasing a key	Reported in paper	NR	ISD	Mean^2^/variance
Gooch et al. (2009)	Visual	Letter	Fixed, 250	40 (8)	4, spatial, location of X	Pressing a response key	Provided by author	Performed	ISD	CV
Gorus et al. (2006)	Visual	Light	Variable, 3,000–6,000	28 (NR)	4, spatial, location of light,	Releasing home key and pressing response key (RT = DT+MT used here)	Provided by author	Performed	IQR	IQR/MD*100
Hogan (2003)	Visual	Arrow	Variable, 1,000–3,000	33 (10)	2, non-spatial, identify the direction of arrow	Pressing a response key	Raw: reported in paper; adjusted: digitised graph	Performed	MD ISD	Median ISD adjusted for median RT
Hultsch et al. (2002)	Visual	Letter	Fixed, 1,000	20 for each level of choice (NR)	2 4 8, spatial, identify location of O	Releasing a home key and pressing a response key (RT used)	Provided by author	Performed	ISD	Purified ISD
Li et al. (2009)	Visual	Circle	Fixed, 1,000	Approx 42 (6 relevant)	2, non-spatial, identify changing colour	Pressing a response key	Provided by author	Performed	ISD	Purified ISD
Martin et al. (2009)	Visual	Light	NR, NR	60 (20)	2 4 8, spatial, identify location	Releasing a home key and pressing a response key (DT used)	Provided by author	Performed	ISD	CV
McAuley et al. (2006)	Visual	Circle	Variable, 600–1,000	40 (10 relevant)	2, spatial, identify location of stimulus changing colour	Pressing a response key	Provided by author	Performed	ISD	CV
Rakitin et al. (2006)	Visual	Digit	Fixed, 500	30 (NR)	4, non-spatial, discriminate between digits (1,2,3,4)	Pressing a response key	Provided by author	Performed	ISD	CV
Shammi et al. (1998)	Visual	Letter	Fixed, 750	180 (10)	2, non-spatial, distinguish between LR and RL (order)	Pressing two keys (L and R) in order determined by stimulus	Estimated from F	NR	ISD	CV
Smulders (1997)	Visual	Digit	Both: Variable 1.520–5.680, Fixed 3.020	Fixed PI: 277; variable PI: 236 (100 per block)	2, non-spatial, distinguish between different digits	Pressing a response key	Estimated from F	Performed	IQR	-
Spirduso & Clifford (1978)	Visual	Light	Fixed, 20,000	50 (NR)	3, spatial, identify the location of stimulus light	Releasing a home key and pressing a response key (DT used)	Reported in paper	NR	Mean of ISDs from 5 blocks of 10 trials	-
West et al. (2002)	Visual	Digit	Fixed, 200	50 (8)	4, non-spatial, discriminate between digits (1,2,3,4)	Pressing a response key	Provided by author	NR	ISD	CV
Williams et al. (2005)	Visual	Letter	Fixed, 500	32 (0 – data from practice block)	2, non-spatial, distinguish between X and O	Pressing a response key	Reported in paper	Performed	-	Purified ISD
Williams et al. (2007)	Visual	Fish	Fixed, 250	20 (10+ mixed conditions)	2, non-spatial, identify direction	Pressing a response key	Reported in paper	Performed	-	Purified ISD

*Note*. CV = coefficient of variation, IIV = intra-individual variability, IQR = inter-quartile range, ISD = intra-individual standard deviation, MD = median, NR = not reported, PI = preparatory interval.

Raw CRT IIV measures obtained from the included studies were either ISD (n = 18) or IQR (n = 2). For most studies adjusted IIV measures was the CV (n = 11; 1 based on IQR). Eight studies used various versions of regression method to partial out effects of RT mean and calculated ISD from the “purified” residuals. For two studies, other measures were available, including age group *R^2^* from regression with median RT entered as a covariate [30], and an unusual conceptualisation of IIV as RT mean^2^/variance, which is equivalent to 1/CV^2^ [42].

Of the 24 studies which considered IIV in CRT in the relevant age groups, 22 contributed to meta-analyses, including 18 studies with data on raw CRT IIV, and 19 studies with data on CRT IIV adjusted for mean CRT. Sufficient data to allow inclusion in the meta-analysis could not be obtained for either CRT IIV measure from two studies [27], [42]. In addition, a study by Shammi et al. [17], which reported sufficient information for raw CRT IIV and was included in a meta-analysis, only provided a verbal account of age effect on mean-adjusted CRT IIV. These studies and their findings are summarised briefly in section 3.2.7. Main analyses of the remaining studies with sufficient CRT IIV data were comparable with those performed for SRT IIV. That is, four meta-analyses were performed: raw IIV in old versus young participants, adjusted IIV in old versus young participants, raw IIV in old versus middle-aged participants, and adjusted IIV in old versus middle-aged participants.

Cochran's Q test was used to assess the heterogeneity between studies in each meta-analysis. There was significant heterogeneity among studies in all four comparisons (all *p*s<.001; *I* ^2^ range 80.56 to 91.45); therefore, random effects method was applied to pool effect sizes.

#### 3.2.1 Raw CRT IIV: old versus young

**Figure 6:**
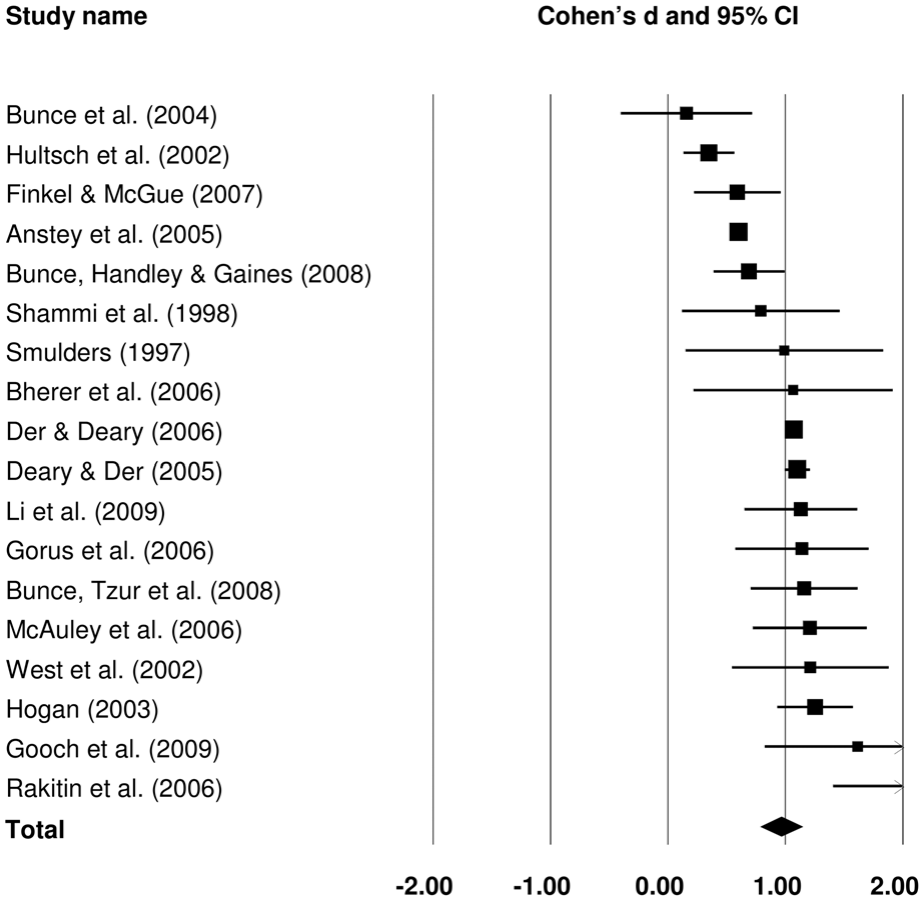
Effect sizes for old versus young comparisons in raw choice reaction time intra-individual variability studies.

Of the identified studies, 18 contributed data to the old versus young comparison of raw CRT IIV. A forest plot summarising individual and pooled effect sizes for this analysis is presented in Figure 6. Cohen's *d* pooled from all studies was 0.960 (*Z* = 10.380, *p*<.001), indicating a large difference between older (more variable) and younger (less variable) groups.

Subgroup analyses were performed to identify potential sources of variance in the effect sizes of different studies. Subgroups were created for sample size and study quality as for SRT. In addition some CRT task characteristics were considered: whether the responses were spatially determined (spatial vs. non-spatial), the number of choices (two vs. four), and whether PI was fixed or variable across task trials. Modality of stimulus presentation was not used here, because all studies employed visual stimuli. Because most raw CRT IIV subgroups included at least five studies, τ^2^ was estimated separately for each subgroup and not pooled. The only exception was the analysis based on sample size, which included subgroups with n = 3 and n = 4.

The results of the subgroup analyses are summarised in Table 5. Studies with smaller samples appeared to produce larger differences between older and younger individuals than studies with medium or large samples. Effect size for studies with large samples was larger than effect sizes for studies with medium samples. However, the differences were not statistically significant (*p* = .169). Effect sizes obtained from studies with more representative samples were slightly lower than from studies with less representative samples, but again, there was no significant between subgroup heterogeneity (*p* = .561). Effect sizes were significantly larger in studies which did not perform trial-level data trimming (*p* = .029). The difference between older and younger participants was more marked in tasks with no spatial component; for example, where a response involved discrimination between different letters or digits, rather than between their spatial position (*p* = .001). The number of choices and PI variability did not explain much between-study variance, with subgroups in each very similar in effect sizes (*p* = .811 and .556, respectively).

**Table 5:** Summary of subgroup analysis results for choice reaction time intra-individual variability studies.

Groups compared	Raw CRT IIV				Mean-adjusted CRT IIV			
	N	ES	Z	p	N	ES	Z	p
All studies	18	0.960	10.380	<.001	19	0.563	6.344	<.001
Sample size				.169^a^				.184^a^
<100	11	1.127	8.317	<.001	10	0.661	4.804	<.001
100–1000	4	0.710	4.010	<.001	6	0.643	4.268	<.001
>1000	3	0.924	5.004	<.001	3	0.259	1.379	.168
Sample representativeness				.561^a^				.135^a^
Not likely	13	1.019	6.859	<.001	14	0.673	3.526	<.001
Likely	5	0.897	6.091	<.001	5	0.348	3.370	.001
Data trimming				.029^a^				.079^a^
Not performed	5	1.132	14.153	<.001	5	0.318	2.219	.026
Performed	13	0.851	8.461	<.001	14	0.678	4.623	<.001
Response type				.001^a^				.464^a^
Non-spatial	9	1.122	20.908	<.001	10	0.654	4.520	<.001
Spatial	9	0.738	7.124	<.001	9	0.494	3.019	.003
Number of possible choices				.811^a^				.965^a^
Two	7	0.988	5.986	<.001	8	0.589	2.642	.008
Four	11	0.942	8.693	<.001	11	0.577	4.642	<.001
PI variability				.556^a^				<.001^a^
Variable	6	1.072	19.733	<.001	6	0.027	0.191	.848
Fixed	9	0.955	4.980	<.001	11	0.923	4.960	<.001
Mean-adjusted IIV measure								.004^a^
CV (based on variance or SD)	-	-	-	-	9	0.271	1.757	.079
CV (based on percentile difference)	-	-	-	-	1	0.262	0.543	.587
ISD on residuals purified of mean	-	-	-	-	8	1.012	6.355	<.001
Other	-	-	-	-	1	0.050	0.117	.907

*Note.* CRT IIV = choice reaction time intra-individual variability, CV = coefficient of variation, ES = effect size (Cohen's d), ISD = intra-individual standard deviation, PI = preparatory interval.

a p value for overall between subgroup heterogeneity.

Bivariate meta-regression was performed with old group age, the number of CRT trials and the PI length as covariates. None of the proposed variables explained a significant amount of variance in raw CRT IIV difference between old and young groups, although some trends could be observed in scatter plots for old group age (positive); see Figure S2.

#### 3.2.2 Raw CRT IIV: old versus middle-aged

**Figure 7:**
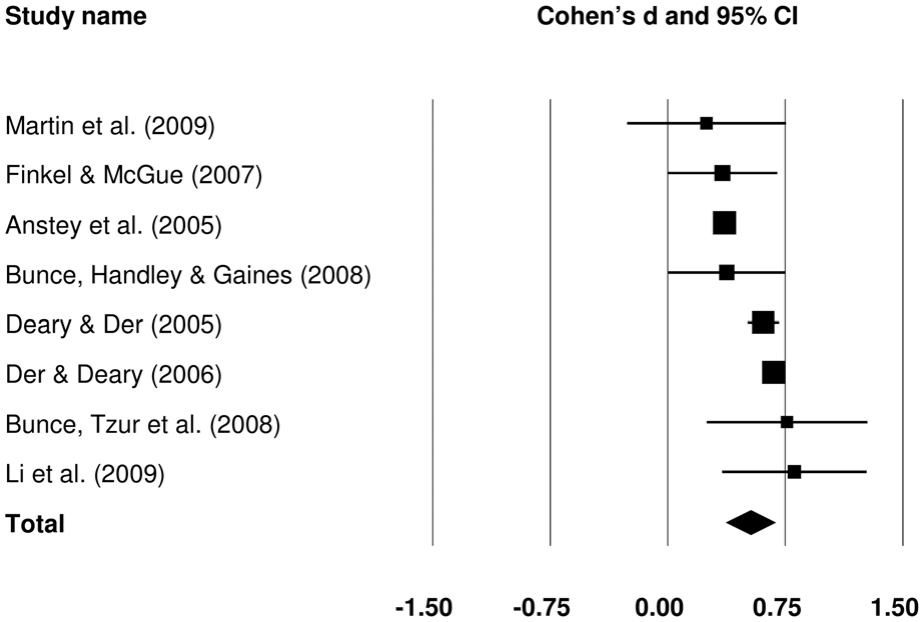
Effect sizes for old versus middle-aged comparisons in raw choice reaction time intra-individual variability studies.

Eight studies provided data on differences in raw CRT IIV between older and middle aged groups (see Figure 7 for a forest plot). The pooled effect size for this comparison, *d = *0.524 (*Z = *6.461, *p*<.001), was medium in magnitude, and lower than the effect size for difference between old and young participants. However, the direction remained unchanged, with older people demonstrating greater IIV. Due to a modest number of studies, sources of heterogeneity were not explored for this comparison

#### 3.2.3 Mean-adjusted CRT IIV: old versus young

**Figure 8:**
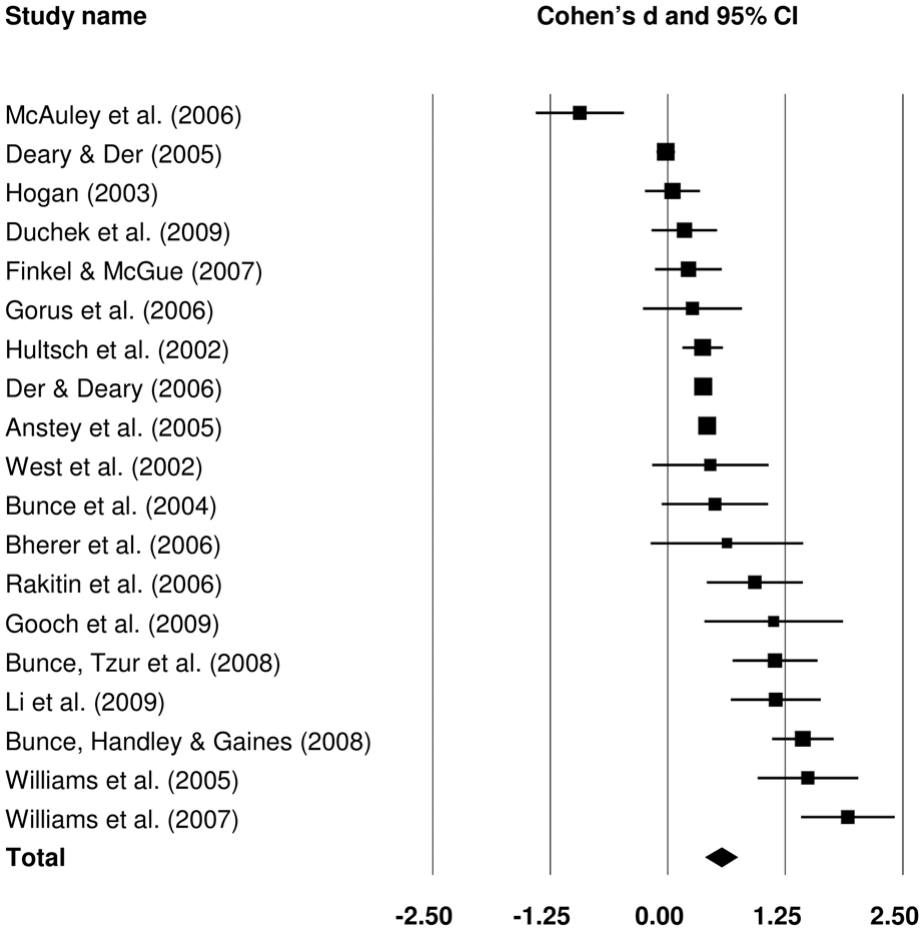
Effect sizes for old versus young comparisons in mean-adjusted choice reaction time intra-individual variability studies.

Differences between old and young groups in the mean-adjusted CRT IIV were pooled from 19 studies (see Figure 8). Older participants showed greater variability, even when it was adjusted for the CRT mean, with a medium effect size, *d* = 0.563 (*Z* = 6.344, *p*<.001). One study provided an estimate which was a clear outlier [48]. When this study was removed, *d* increased to 0.632.

Subgroup analyses were performed, including subgroups considered in raw CRT IIV comparison (sample size, sample representativeness, trial-level data trimming, spatial nature of responses, the number of choices, and ISI variability) with the addition of mean-adjusted CRT IIV measure, including CV, ISD from purified residuals, and other. For analyses of subgroups based on sample size and IIV measure, pooled τ^2^ was used; all other subgroups comprised more than five studies and so, separate τ^2^ were estimated for each.

The outcome of subgroup analyses are presented in Table 5. There was little difference in effect sizes obtained from small and medium studies. The difference between older and younger participants in the largest sample subgroup was smaller than either in small or medium samples, but there was no significant variance between the groups (*p = *.184). The effect size was larger for the subgroup with less representative samples, but again no significant difference was found between the two groups (*p = *.135). Whether individual trial data were trimmed or not appeared to explain a degree of between study heterogeneity; the effect size was larger with trimming than without (although, this was only a trend, *p = *.079). The difference between older and younger participants was slightly smaller in tasks concerned with spatial location of stimuli rather than their formal discrimination. However, there was no significant heterogeneity between these two groups of studies (*p* = .464). The number of choices also did not appear to explain much heterogeneity (*p = *.965). There was a marked difference in effect sizes between subgroups based on PI variability. When PIs varied, the effect size for old-young difference in mean-adjusted CRT IIV was negligible (0.027). However, with fixed ISI, the effect size was large and significant (0.923). This large difference was statistically significant (*p*<.001). Further investigation of this finding revealed that median PI length was greater for variable (2,000 ms) than for fixed PIs (500 ms). Finally, when different measures of IIV were considered, the effect sizes obtained from studies using purified residuals to calculate ISD were notably larger than effect sizes from studies using CV (either based on ISD or percentile differences) or other measures (*p*<.001).

Meta-regression was performed to assess whether age of the old group, the number of trials or the length of PI could explain some of the between-study heterogeneity. Effect sizes were smaller at larger PIs (*B = *−0.170, *se = *0.081, *p = *0.035), but there was no significant effect of either the age of the older group or the number of CRT trials.

#### 3.2.4 Mean-adjusted CRT IIV: old versus middle-aged

**Figure 9:**
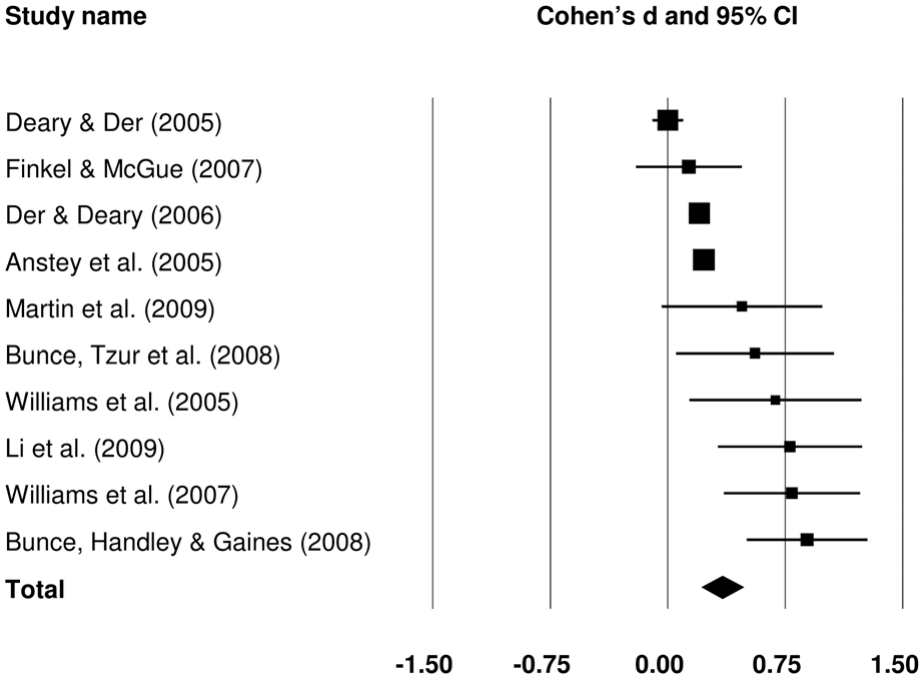
Effect sizes for old versus middle-aged comparisons in mean-adjusted choice reaction time intra-individual variability studies.

Ten studies contributed to the comparison of mean-adjusted CRT IIV between old and middle-aged groups (forest plot can be seen in Figure 9). The pooled effect size was small in magnitude, *d* = 0.344 (*Z* = 4.979, *p*<.001) and revealed that older participants demonstrated greater mean-adjusted IIV in CRT than did middle-aged individuals.

#### 3.2.5 Attenuation of age group difference in effect sizes by adjusting CRT IIV for CRT mean

Effect sizes obtained from the four meta-analyses performed on the CRT IIV data, showed a similar pattern to that observed in SRT IIV data. That is, the differences were larger for greater age difference and larger if CRT IIV was not adjusted for CRT mean. The degree of effect size attenuation by adjusting CRT IIV measures for CRT mean was investigated as before: pooled effect sizes were obtained from studies which provided data on both raw and mean-adjusted CRT IIV within both age group comparisons (old versus young and old versus middle-aged). Overall, 16 studies were used for the calculation of effect size for old versus young differences and eight studies contributed data to the old versus middle-aged comparisons. If a mean-adjusted CRT IIV measure is used, the effect sizes are attenuated by 52.4% in old versus young comparison (raw CRT IIV *d* = 0.967; mean-adjusted CRT IIV *d* = 0.460), and by 46.2% in old versus middle-aged comparison (raw CRT IIV *d* = 0.524; mean-adjusted CRT IIV *d* = 0.282).

#### 3.2.6 The number of possible choices in CRT

Some studies provided data on more than one version of CRT task in terms of the number of possible choices. However, to ensure the independence of effect sizes included in meta-analyses, only one level of choice was selected for the main analysis. Three studies reported data from 2-, 4-, and 8-choice RT tasks, with a further two studies reporting data on both 2-, and 4-choice RT tasks. From these, only the 4-choice RT data were included in the analyses performed previously. To allow more of the available data from these studies to be used, additional analyses were performed whereby a separate estimate of effect size was obtained for both mean-adjusted and raw measures of CRT IIV for 2 and 4-choice RT (there were only three studies which reported data on 8-choice task, rendered insufficient for meta-analysis).

There were altogether 11 studies which provided data on raw 2-choice RT IIV, and 12 on raw 4-choice RT IIV. The effect sizes estimated from these were 0.925 (*Z = *8.574, *p*<.001) and 0.893 (*Z = *8.292, *p*<.001), respectively. There were 13 studies that provided mean-adjusted measures of 2-choice RT IIV and 11 studies with mean-adjusted 4-choice RT IIV data. Effect sizes for old-young difference in these were 0.713 (*Z = *4.459, *p*<.001) and 0.577 (*Z = *4.642, *p*<.001), indicating a somewhat smaller difference in 4-choice than in 2-choice RT task.

#### 3.2.7 Evidence from CRT IIV studies not included in meta-analyses

Fozard et al. [42] reported on CRT IIV of participants in the Normative Aging Study. They noted that CRT ISDs were larger for older than middle-aged or younger groups. An ANOVA performed on all age groups included in the sample revealed a significant effect of age. Fozard et al. also considered a measure of CRT IIV adjusted for mean, 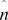, which represents mean^2^/variance ratio (with greater values indicating less variability). There was a significant effect of age on this measure, but post-hoc analysis revealed that only the differences between younger and middle-aged group were significant. However, a significant negative correlation between 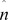 and age indicated that RT IIV increases with age (and does so to a greater extent that mean RT).

Spirduso and Clifford [27] measured raw CRT ISD of younger and older participants engaging in different levels of physical activity. For non-active groups, mean CRT ISDs were greater for older (44 ms) than for younger individuals (39 ms). However, the difference was much larger between active and non-active groups, than between older and younger groups. Indeed, activity level, but not age, had a significant effect on CRT ISD.

Finally, Shammi et al. [17] considered both raw and mean-adjusted measures of CRT IIV. Data provided for the former were sufficient for inclusion in the meta-analysis; however, only a verbal account of the results are provided for the CV. Shammi et al. noted that the difference between older and younger participants was only significant when IIV was conceptualised as a raw ISD. When CV was used, the effect of age was not significant.

### 3.3 Assessing publication bias

The file-drawer problem [55], that is an under-representation of non-significant studies which are not published, poses a genuine threat to the estimated effect size in meta-analytical procedures. Therefore, evidence of publication bias in the extracted data was assessed. This was done for old-young comparisons only, because there were greater numbers of studies included in those than in the old versus middle-aged comparisons. Three common procedures were adopted to assess publication bias. Firstly, the fail-safe N was calculated. This method estimates the number of hypothetical unpublished studies with an average effect size of 0 which, if they were included in meta-analysis, would increase the p value for the meta-analysis to borderline significance (*p* = .05). In other words, fail-safe N indicates how robust a meta-analytic result is to publication bias. If the overall meta-analytic p value was increased to .05 (i.e. just significant) by inclusion of only a few null results, then there is a substantial threat of file-drawer effect. Secondly, funnel plots were visually inspected and checked for symmetry. Funnel plots allow graphical assessment of the association between effect sizes and standard errors of studies included in a meta-analysis. The premise behind them is that larger studies (with smaller standard errors) provide more precise estimates of the effect size. Therefore, the effects will be scattered for small studies and become more concentrated around the “true” effect size for larger studies, creating a funnel shape. If there is no bias, the funnel plot will be symmetrical; deviations from symmetry indicate probable publication bias. Finally, trim and fill technique proposed by Duval and Tweedie [56], [57] was adopted to re-calculate “corrected” effect sizes taking into account studies potentially missed due to publication bias. .

The fail-safe N for raw and mean-adjusted SRT IIV were 1,503 (equivalent to approximately 116 missing studies for each observed study) and 410 (approximately 32 missing studies for each observed study), respectively. For raw CRT IIV, the fail-safe N was 4,109 (228 missing studies per each one observed), and for mean-adjusted CRT IIV the number of non-significant studies which would nullify the effect was 1,236 (65 per each one observed). Overall, the fail-safe Ns were very large for all comparisons considered.

**Figure 10:**
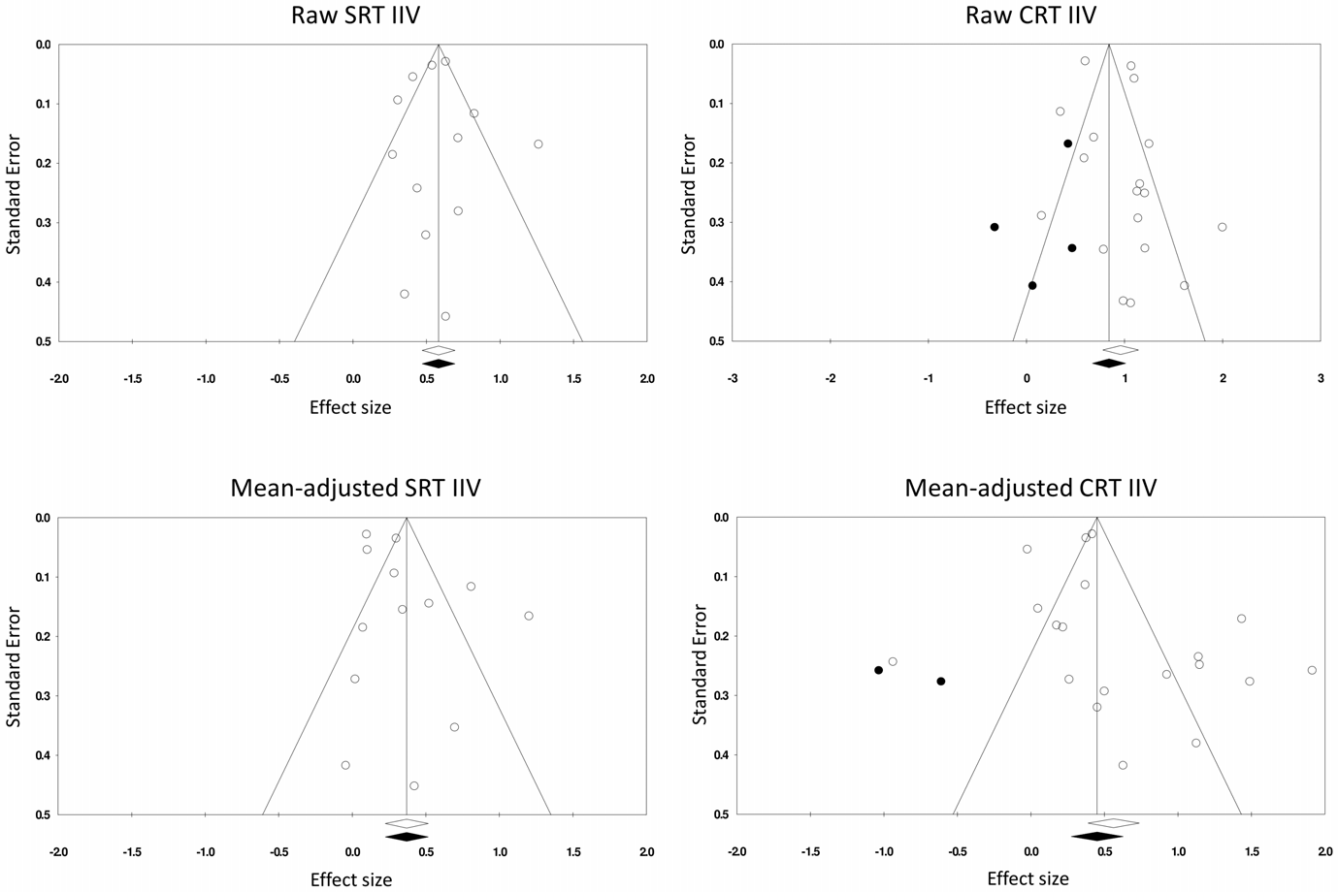
Funnel plots showing little evidence of publication bias of raw or mean-adjusted simple reaction time intra-individual variability studies (left panel), with some bias apparent among studies of raw and mean-adjusted choice reaction time intra-individual variability (right panel). Open symbols = actual studies, filled symbols = “missing” data points identified by the trim and fill procedure (Duval & Tweedie, 2000a,b).

Funnel plots of effects based on raw and mean-adjusted SRT IIV and CRT IIV are presented in Figure 10. For both SRT IIV measures, studies are centred around the overall effect size. The plots do not reflect the usual funnel shape, but departures from symmetry are not marked. There are relatively few studies with large standard errors; however, they are under-represented among both positive and negative poles. Trim and fill procedure did not identify any “missing” points and the overall effect size remained unchanged for both raw SRT IIV and SRT IIV adjusted for mean SRT. Funnel plots for CRT IIV were clearly non-symmetrical. For both raw and mean-adjusted CRT IIV, trim and fill procedure (under random effects model) identified a number of “missing” studies. Consequently, the pooled effect sizes were reduced from 0.960 to 0.842 for raw CRT IIV and from 0.563 to 0.451 for mean-adjusted CRT IIV.

## Discussion

This meta-analysis consistently found greater IIV at older than younger ages. The finding was consistent in comparisons performed for both SRT and CRT, and in comparisons between different age groups (old versus young as well as old versus middle-aged). Effect sizes were larger for CRT than SRT, supporting the notion that age effects are more pronounced in more demanding tasks [4]. SRT and CRT differ in that CRT requires an additional processing step: i.e., response selection. An obvious explanation for greater difference between older and younger participants in IIV in CRT than SRT is that an increased number of mental operations provides more scope for variability. Perhaps the effects of age on variability occur at different steps involved in a task and are additive. However, neither of the tasks considered in this meta-analysis were thought to involve executive processes, such as planning, problem solving, or inhibition. Therefore a greater age effect in the more difficult task (CRT) could not be attributed to age-related increases in executive control fluctuations as was the case in [21].

Our investigation of CRT difficulty by comparing effect sizes obtained from CRT tasks involving 2 or 4 stimuli did not reveal a similar pattern, however. When the number of possible choices was considered in subgroup analysis, no significant differences were found between the two groups for either raw or mean-adjusted CRT IIV. When separate meta-analyses were performed on all studies that provided data on 2- and 4-choice RT IIV, we obtained slightly smaller effects for the more difficult task with 4 possible choices. Admittedly, the differences were rather small and further empirical investigations are required to test whether the number of possible choices in a CRT task has an effect on age differences in CRT IIV.

The finding of greater effect sizes in the old versus young comparisons than in the old versus middle-aged comparisons was expected given that age-related increases in RT IIV occur throughout adulthood and that IIV reaches a lifetime minimum in the early 20s [2]. However, the fact that the pattern was apparent in both SRT and CRT was somewhat surprising. Earlier accounts suggested that CRT IIV increases throughout the adult age range, whereas SRT IIV remains relatively stable or even decrease slightly in the early 20s [2]. One explanation may be that many of the middle-aged groups included individuals up to the age of 60. Even if RT IIV increases only in old age (defined here as 60 years and above), it is reasonable to assume some increases can be observed among individuals just below this arbitrary cut-off. Therefore, the middle-aged groups might have contained both younger middle-aged individuals (whose SRT IIV are still stable) and older middle-aged participants (whose SRT variability might have already started increasing). The presence of such older and more variable individuals would inflate the mean SRT IIV of the middle-aged group. A relatively small number of studies in this review precluded further analyses to test this hypothesis, such as a meta-regression with mean age as a covariate. In any case, the pattern of smaller differences between the middle and old group than between the young and old group indirectly suggests that whereas older adults have markedly more variable RTs than young adults, some increase in RT IIV occurs already before the age of 60.

An alternative explanation for the finding may be in terms of cohort effects. Given that the age gap between the groups of interest in this review could be as large as 40 years and all studies were cross-sectional, it is possible that cohort effects created (or at least added to) the observed younger-older differences in RT IIV. However, studies included in this review spanned a few decades and all found similar pattern of age effects. In fact, even in studies published 20 [33] or even 60 years ago [49], the same pattern of age effects is apparent: older adults are significantly more variable in RTs than younger adults, rendering the cohort effect explanation unsupported.

Another finding of this review was that greater RT IIV among older individuals was found regardless of whether an IIV measure was or was not adjusted for RT mean. Effect sizes for mean-adjusted IIV were smaller than for raw IIV measures, implying that some (but not all) of the age-related increase in RT IIV is mediated by the slowing of RTs with age. One explanation for this finding is that there might be different “components” of RT IIV. The total age-related increase in IIV may be a combination of greater variability which shares common variance with the slowing of responding (and is therefore removed by adjusting IIV for mean RT) and greater variability due to other causes. RT variability is somewhat “constrained” by the mean in the bottom part of the distribution (i.e. shortest RTs). Considering that there is minimum RT (it cannot be less than 0, and researchers often adopt a theoretical minimum RT of 100 ms or 150 ms thought to be the minimum amount of time required to execute a motor response) but not a maximum limit, an increase in RT mean could be expected to have an effect on the shape of the distribution whereby it becomes less positively skewed. In other words, if all IIV was due to mean RT increase, then one could expect more increase in the number of very short responses (which are more constrained by short overall RTs) than very long responses. There is no reason to expect a disproportionate increase in very long RTs with increasing RT mean, as these are theoretically possible at any level of overall speed of responding. However, some authors note that such an increase in very long RTs (sometimes termed attentional blocks) is indeed observed among older adults [21], [24]. It was not the focus of this meta-analysis to separately investigate slow and fast portions of the RT distribution; therefore, the proposition of different effects of mean RT on these remains untested. However, the finding of the present review is consistent with the view that both general and specific variability-producing influences may be at play at older ages [24]. Therefore, it is possible that age-related increase in IIV comprises greater variability brought about by larger mean RT as well as a larger number of very long RTs with other underlying mechanisms (and so, not removed by the adjustment for RT mean). This possibility warrants further empirical investigation.

Papers published since the review had been performed, largely support its findings. ICV has been reported to be larger at older ages across a range of RT tasks [58]–[61]. Deary et al. [59] compared the performance of 150 participants in three age groups (18–25, 45–60, and 61–80) on two different SRT and CRT tasks. The effect sizes ranged from very small (*d = *0.01 for old versus middle-aged SRT IIV) to 1.96 (for old versus young in CRT IIV). The pattern of effects was similar to the one from our meta-analyses—the effects are larger for old versus young rather than old versus middle-aged comparisons, and larger for CRT than SRT.

A study that considered both raw and mean-adjusted RT IIV [62] showed that, among adults aged between 18 and 75 years, CRT ISD and CRT CV increase with age. The effect of age was not significant for SRT IIV or SRT CV, but this could be due to a relatively small sample of older adults in that study (n = 35 for ages 60 years and above).

Another study [58] showed that older participants (mean age = 71.24) have more variable performance than younger participants (mean age = 21.29) on both traditional RT and on a driving task. The correlations for 2-choice RT and 4-choice RT were .46 and .66, respectively, translating into Cohen's *d*s of 1.04 and 1.80.

### 4.1 Explaining heterogeneity

Subgroup analysis and meta-regression identified a few potential sources of between-study heterogeneity. However, these were rarely replicated across different comparisons; i.e., few were common for all raw SRT IIV, mean-adjusted SRT IIV, raw CRT IIV and mean-adjusted CRT IIV studies.

Sample size appeared to explain some heterogeneity, although it only approached significance in mean-adjusted SRT IIV. Moreover, only in studies of adjusted CRT was the pattern as one could expect; that is, effect sizes were larger for small and medium studies than for large studies. However, this lack of a pattern of larger effect for smaller studies may simply reflect a relatively low “publication” bias in the present review, as it included effect sizes which were not even reported in their original publications. On the contrary, the degree of sample representativeness produced similar results in all comparisons, with effect sizes larger for less representative samples. Although not reaching statistical significance other than in mean-adjusted SRT IIV, sample representativeness led to a pattern consistent across all four measures of RT IIV. This is not surprising, given that many of non-representative samples in studies included in this review were selected specifically to test old-young differences and some young groups consisted primarily of students. Purposive samples of older and younger participants are likely to be more different than similar age groups taken from the population. If different age groups are recruited from different populations, it is likely that the real age effects are exaggerated.

Trial-level trimming did not explain much between study heterogeneity. There was no significant difference between subgroups based on trimming in either raw or mean–adjusted SRT IIV. For raw CRT IIV studies, trial-level trimming was associated with larger effect sizes; however, a trend for the opposite pattern was observed in mean-adjusted CRT IIV. Therefore, no clear conclusion can be drawn from this subgroup analysis. Since trimming often involves excluding aberrant RTs thought to result from accidental responses (very short) or distractions/loss of concentration (very long), this procedure improves the precision of estimates of RT IIV. It follows that with a better measure, more precise effects are obtained, which should produce a clearer picture of the actual age differences in RT IIV. That is, the effect should be larger in studies which performed the trimming. On the other hand, trimming decreases the estimates of variability. Given that RT of older adults tend to include more very long responses [21], [24], it is likely to reduce the IIVs of older people more than those of younger individuals, especially if the same cut-offs are used for all individuals and a greater proportion of trials are removed in older groups. Consequently, the effect size would be *smaller* than when no trimming is performed. These opposing forces could act together to render effects of trial-level trimming non-significant.

For studies of adjusted SRT and CRT, different measures of IIV were considered and significant heterogeneity between subgroups based on the IIV measures was found for CRT IIV. Age effects were larger for IIV obtained from purified residuals, than for either CV or other methods. Although CV is often criticised as a method of adjusting RT ISD for mean RT, it does provide adjustment of each individual's ISD for his or her own mean RT. On the contrary, purified residuals are typically obtained from a regression line which is fitted to all participants, and are rarely adjusted for individual speed of responding. Therefore, CV may actually provide a more precise measure of mean-adjusted IIV than ISD of purified residuals.

There was a trend toward larger effect sizes in studies of SRT IIV which used visual rather than auditory stimuli. Given that RTs are usually shorter in response to auditory than visual stimuli [18], this effect may reflect greater IIV among older adults brought about by their longer RTs. The lack of a similar trend with mean-adjusted SRT IIV measures provides some support for this explanation. However, since there was only one study which used an auditory stimulus and provided SRT IIV, this explanation remains tentative.

The comparisons of procedural factors for studies of CRT, included the response type, number of possible choices, and PI variability. The finding of larger effect sizes when a response was non-spatial (statistically significant for raw CRT IIV) is a novel one. A possible explanation for the effect may be that tasks with spatial components are largely perceptual rather than cognitive, hence requiring less processing and leaving less scope for age-related variability. The remaining procedural factors did not explain heterogeneity in raw CRT, although for mean-adjusted CRT effects were larger with fixed rather than variable PIs. Given that this effect did not replicate across the two CRT IIV measures, the apparent differences should be treated with caution. However, it is worth noting the magnitude of the difference between the two subgroups: 0.027 for variable (0.172 with [48] removed) and 0.923 for fixed PI. This large difference is in the opposite direction to that expected from the existing literature. Given that older adults seem particularly affected by encountering very short PIs among longer ones [19], the old-young IIV difference should be larger, not smaller, in studies adopting variable PIs. A further investigation of the finding revealed that the PI variability was confounded with PI length, in that fixed PIs were notably shorter than variable PIs. Therefore, it may not be PI variability per se, but rather the length of the interval used that is related to age differences in CRT IIV. The finding would be worth exploring further in future empirical studies.

Finally, results from meta-regression analyses provide some candidate covariates. However, none replicate across the four RT measures considered in this review. Given the relatively small number of studies included in the meta-regression analyses, some null findings could be due to insufficient power. Old group age was related to the old-young effect sizes in raw and adjusted SRT, with the differences larger for older old groups (i.e. old groups with higher mean age). The mean age of old group, however, did not predict effect sizes in CRT. This pattern of findings is in contrast to what could be expected from earlier investigations, suggesting that CRT IIV start to increase with age earlier than SRT IIV e.g. [2]. The value of PI was associated with effect sizes in raw SRT and adjusted CRT, with smaller effects at longer PIs. The number of trials did not predict the magnitude of old-young difference for any IIV measure, which was again contrary to the expectation.

### 4.2 Publication bias

None of the three methods of publication bias assessment revealed bias among studies of either raw or adjusted SRT IIV. There were relatively few studies with large standard errors, but they were “missing” equally from higher as well as lower ends of the effect size distributions. Some publication bias was detected among studies of CRT IIV, however. Funnel plots presenting both raw and mean-adjusted CRT IIV studies were asymmetrical and more studies than expected had large and positive effect sizes (indicating greater variability among older groups). Trim and fill procedure led to a reduction of the pooled effect sizes for both sets of studies.

Since effect sizes tend to be larger for raw than adjusted IIV measure, selective publication of only the results obtained from raw measures when adjusted IIV shows no difference between young and old, might occur. However, given that in this review attempts were made to include data on both measures from each relevant study, this explanation is not likely to be accurate. It should also be noted that the “missing” studies identified by the trim and fill procedure largely fall within the realm of negative effect sizes. In other words, the procedure implies that most of the missed studies rather than finding no difference between the age groups, would find younger groups to have greater variability. This scenario is highly unlikely, and so the extent of the publication bias suggested may be overestimated.

### 4.3 Strengths and limitations

There were a number of strengths of this review. Firstly, it was a large, thorough and comprehensive review of published studies on SRT IIV and CRT IIV. Secondly, strict inclusion and exclusion criteria ensured that the reviewed studies were acceptably similar in terms of RT tasks and participant groups. Thirdly, this review included all studies from which the relevant data could be obtained, even if age differences in RT IIV were not the main focus of a study. Therefore, it can be expected that effects of publication bias would be reduced. Finally, attempts were made to obtain both raw and mean-adjusted measures from all included studies and this has been successfully achieved for a large proportion of the identified studies.

Among the limitations is the cross-sectional design of the included studies. The number of longitudinal investigations into RT IIV was scarce, and the length of follow-up did not allow comparison of IIV at young, middle and older age. An obvious problem associated with cross-sectional investigation is the potential confounding of age differences with cohort effects. However, as already mentioned, given that age group differences are observed in studies carried out a few decades ago as well as those more recent, there are sound grounds for concluding that greater IIV in older than younger ages is a genuine effect.

Another limitation of the review is that despite the attempts to keep studies similar by controlling the nature of RT tasks administered, there were notable procedural differences between those that were included. Studies differed in the type of stimuli used, the PI, the number of trials, and also in the treatment of data (including data preparation prior to analysis). These resulted in significant between-study heterogeneity, yet no clear sources of it could be identified. We have addressed this issue by using random effects models in order to provide more accurate estimates of effect sizes in light of the between-study heterogeneity. One striking finding was that despite the heterogeneity, there was commonality – the vast majority of the studies reported an age effect in RT IIV in the expected direction (i.e., with older groups showing greater RT IIV). Therefore, although the magnitude of the effect varied across the studies, the direction of it did not, suggesting that older-younger difference in RT IIV is relatively robust to procedural differences.

A final limitation of this systematic review is the limited control of the comparability of age groups in terms of education, general mental ability, and health. A review relies on the information other authors had considered and these vary from study to study. Studies that compared their age groups on education commonly find either no difference or more education in younger than older groups. It is not clear whether more education in younger groups could explain some of their superior performance, not least because younger groups were educated at times when the national minimum ages for leaving full time education and the normal age for completing education were higher. Moreover, although the majority of studies attempted to ensure that participants, especially in the older group, were healthy and medication-free, not all studies report this. Among those that do, there are marked differences in how healthy status was defined and ascertained. These differences could all contribute to the relatively poorer performance (i.e. greater variability) in RT performance of older adults and could be investigated as topics in themselves now that we have established the basic effect in these meta-analyses.

### 4.4 Summary

To summarise, this review established that RT IIV is larger in older than younger individuals. The difference between old and young groups was larger than that between old and middle-aged groups, suggesting that increase in RT IIV is not limited to old age, but occurs already in mid-adulthood. Age effect on RT IIV was also larger in CRT than SRT, but not for different number of choices in CRT tasks. The differences in RT IIV between older and younger adults were larger for raw than RT mean-adjusted measures of variability, indicating that some of the increase in IIV with age shares common variance with increases in mean RT. However, this finding does not support claims that all of the observed increase in RT IIV can be explained by slowing of responses with age. Procedural factors did not account for much of the between-study heterogeneity, potentially due to a modest number of studies in most identified subgroups. However, the direction of effect sizes across studies and subgroups pointed towards a larger RT IIV in older individuals.

## Supporting Information

Appendix S1 **PRISMA checklist.**
(DOC)

Appendix S2 **Search terms used for each database.**
(DOCX)

Figure S1 **Scatterplots of effect sizes for old-young differences in raw (left panel) and mean-adjusted (right panel) simple reaction time intra-individual variability (SRT IIV) and the three covariates: old group age mean (top panel), number of trials (middle panel) and the length of preparatory interval (bottom panel).** Circles are proportional to study weights.
(TIF)

Figure S2 **Scatterplots of effect sizes for old-young differences in raw (left panel) and mean-adjusted (right panel) choice reaction time intra-individual variability (CRT IIV) and the three covariates: old group age mean (top panel), number of trials (middle panel) and the length of preparatory interval (bottom panel).** Circles are proportional to study weights.
(TIF)
