# Supplementary material for: Age Differences in Intra-Individual Variability in Simple and Choice Reaction Time: Systematic Review and Meta-Analysis
Source: PLoS One. 2012 Oct 11;7(10):e45759. doi: 10.1371/journal.pone.0045759 (PMC3469552; doi:10.1371/journal.pone.0045759)
Supplement: Appendix S2 — Search terms used for each database. (DOCX) [file pone.0045759.s002.docx]

**Appendix S2. Search terms used for each database**

**PsycINFO**

The first, main search of PsycINFO database was done through EBSCO Host gateway, using advanced search facility of all available fields. The search terms used were:

((reaction time) OR (cognition) OR (processing speed) OR (response speed))

AND

(variability OR inconsistency OR lability OR instability).

The update search was performed via OvidSP using the following search terms:

1. reaction time.mp.

2. exp Reaction Time/

3. cognition.mp.

4. exp Cognition/

5. processing speed.mp.

6. exp Cognitive Processing Speed/

7. response time.mp.

8. 1 or 2 or 3 or 4 or 5 or 6 or 7

9. variability.mp.

10. inconsistency.mp.

11. lability.mp.

12. instability.mp.

13. standard deviation/

14. 9 or 10 or 11 or 12 or 13

15. 8 and 14

16. limit 15 to yr="2008 - 2009"

**MEDLINE and EMBASE**

Searched via Ovid SP gateway with the same search criteria used for both databases:

1. reaction time.mp.

2. processing speed.mp.

3. response speed.mp.

4. cognition.mp.

5. variability.mp.

6. inconsistency.mp.

7. lability.mp.

8. instability.mp.

9. 1 or 2 or 3 or 4

10. 5 or 6 or 7 or 8

11. 9 and 10

**Web of Science**

Accessed via ISI Web of Knowledge Platform and searched using the following search strategy:

1. Topic=(reaction time) OR Title=( reaction time)

2. Topic=(processing speed) OR Title=(processing speed)

3. Topic=(response speed) OR Title=(response speed)

4. Topic=(cognition) OR Title=(cognition)

5. Topic=(variability) OR Title=(variability)

6. Topic=(inconsistency) OR Title=(inconsistency)

7. Topic=(lability) OR Title=(lability)

8. Topic=(instability) OR Title=(instability)

9. 1 OR 2 OR 3 OR 4

10. 5 OR 6 OR 7 OR 8

11. 9 AND 10
