# Supplementary figures and images for: Age Differences in Intra-Individual Variability in Simple and Choice Reaction Time: Systematic Review and Meta-Analysis
Source: PLoS One. 2012 Oct 11;7(10):e45759. doi: 10.1371/journal.pone.0045759 (PMC3469552; doi:10.1371/journal.pone.0045759)

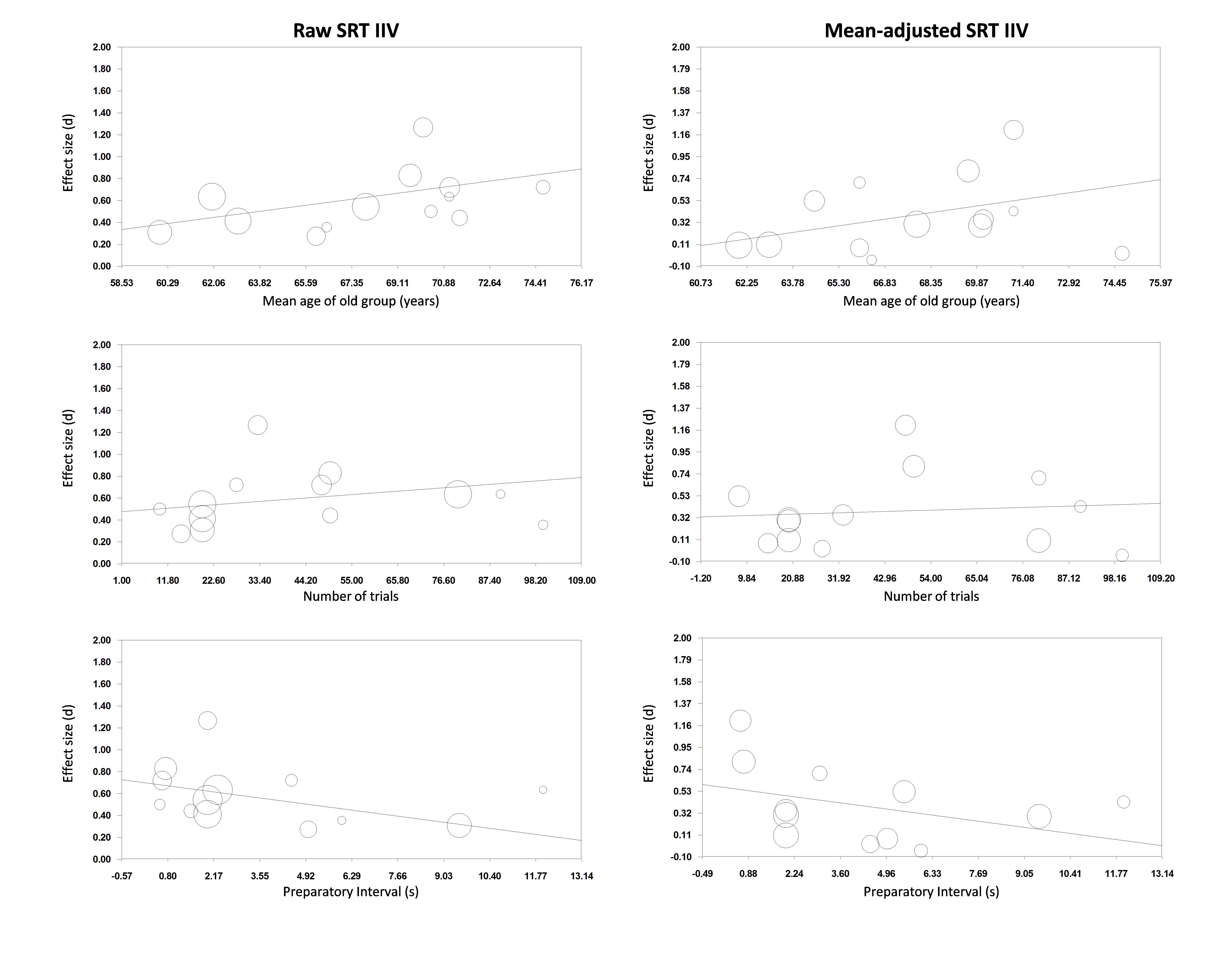

Supplement: Figure S1 — Scatterplots of effect sizes for old-young differences in raw (left panel) and mean-adjusted (right panel) simple reaction time intra-individual variability (SRT IIV) and the three covariates: old group age mean (top panel), number of trials (middle panel) and the length of preparatory interval (bottom panel). Circles are proportional to study weights. (TIF) [file pone.0045759.s003.tif]

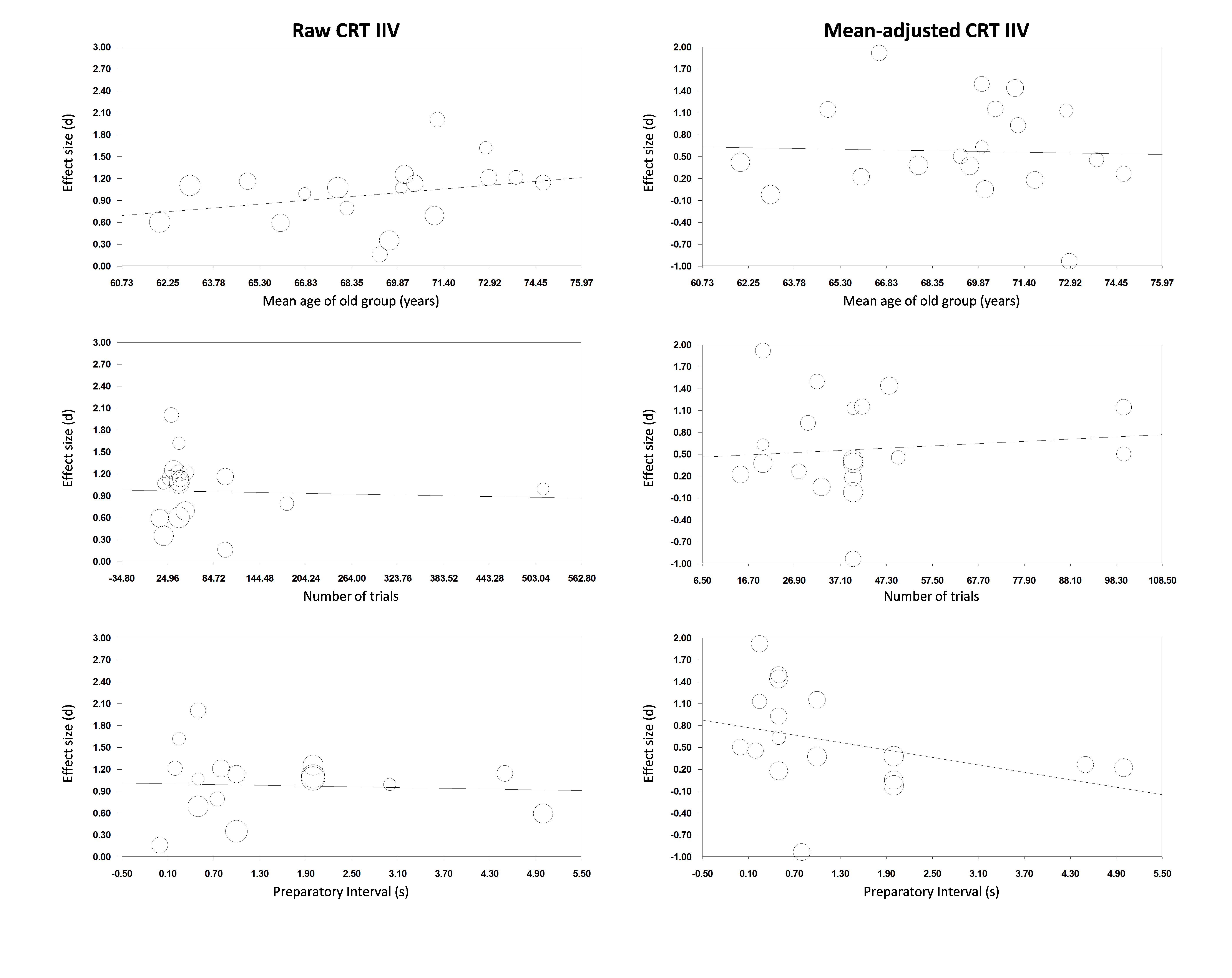

Supplement: Figure S2 — Scatterplots of effect sizes for old-young differences in raw (left panel) and mean-adjusted (right panel) choice reaction time intra-individual variability (CRT IIV) and the three covariates: old group age mean (top panel), number of trials (middle panel) and the length of preparatory interval (bottom panel). Circles are proportional to study weights. (TIF) [file pone.0045759.s004.tif]
